# A strategy for evaluating pathway analysis methods

**DOI:** 10.1186/s12859-017-1866-7

**Published:** 2017-10-13

**Authors:** Chenggang Yu, Hyung Jun Woo, Xueping Yu, Tatsuya Oyama, Anders Wallqvist, Jaques Reifman

**Affiliations:** Department of Defense Biotechnology High Performance Computing Software Applications Institute, Telemedicine and Advanced Technology Research Center, U.S. Army Medical Research and Materiel Command, Fort Detrick, Fort Detrick, MD 21702 USA

**Keywords:** Gene set enrichment analysis, Pathway analysis, Method evaluation

## Abstract

**Background:**

Researchers have previously developed a multitude of methods designed to identify biological pathways associated with specific clinical or experimental conditions of interest, with the aim of facilitating biological interpretation of high-throughput data. Before practically applying such pathway analysis (PA) methods, we must first evaluate their performance and reliability, using datasets where the pathways perturbed by the conditions of interest have been well characterized in advance. However, such ‘ground truths’ (or gold standards) are often unavailable. Furthermore, previous evaluation strategies that have focused on defining ‘true answers’ are unable to systematically and objectively assess PA methods under a wide range of conditions.

**Results:**

In this work, we propose a novel strategy for evaluating PA methods independently of any gold standard, either established or assumed. The strategy involves the use of two mutually complementary metrics, recall and discrimination. Recall measures the consistency of the perturbed pathways identified by applying a particular analysis method to an original large dataset and those identified by the same method to a sub-dataset of the original dataset. In contrast, discrimination measures specificity—the degree to which the perturbed pathways identified by a particular method to a dataset from one experiment differ from those identifying by the same method to a dataset from a different experiment. We used these metrics and 24 datasets to evaluate six widely used PA methods. The results highlighted the common challenge in reliably identifying significant pathways from small datasets. Importantly, we confirmed the effectiveness of our proposed dual-metric strategy by showing that previous comparative studies corroborate the performance evaluations of the six methods obtained by our strategy.

**Conclusions:**

Unlike any previously proposed strategy for evaluating the performance of PA methods, our dual-metric strategy does not rely on any ground truth, either established or assumed, of the pathways perturbed by a specific clinical or experimental condition. As such, our strategy allows researchers to systematically and objectively evaluate pathway analysis methods by employing any number of datasets for a variety of conditions.

**Electronic supplementary material:**

The online version of this article (10.1186/s12859-017-1866-7) contains supplementary material, which is available to authorized users.

## Background

Researchers commonly identify genes that are up- or down-regulated under biological conditions of interest to understand the molecular mechanisms underlying biological processes. However, genes almost always act cooperatively rather than independently. Consequently, methods that analyze the functional contributions of genes should properly take such interactions into account. Biological pathways—the “wiring diagrams” describing the interactions of gene products and other biomolecules as well as their regulatory relationships corresponding to certain biological processes—provide the means to characterize this cooperative nature of gene expression. Pathway analysis (PA) algorithms, combined with large molecular interaction databases, allow for the processing of high-throughput genomic (or proteomic) data, such as those from gene expression microarray or RNA-seq experiments, and the rank ordering of significantly perturbed (up- or down-regulated) pathways associated with an experimental (or clinical) condition [1, 2]. The resulting list of ordered pathways may provide insights into the underlying molecular mechanisms of the experimental condition and help generate testable hypotheses concerning disease biomarkers or therapeutic targets [3].

Rapid technological advancements during the past decade in our ability to generate and collect high-throughput genomic data have spurred the development of a large number of PA methods. A review article in 2009 reported 68 such methods [4], and many more have been developed since [2, 5, 6]. An important step that must precede the use of these PA methods is to evaluate their performance and reliability, using well-characterized datasets where the pathways associated with the tested conditions, i.e., the perturbed pathways, are known in advance. However, this knowledge of ‘ground truths’ (or gold standards) is sometimes incomplete and often unclear [7, 8].

To address the lack of a gold standard, researchers have developed numerous strategies, many of which are centered on defining such ‘true answers.’ One approach utilizes simulated gene expression data to create a hypothetical gold standard [9–11], where a particular set of pathways is assumed to be perturbed and the expression levels of the constituent genes are changed accordingly. The drawback of this approach, however, is that because we do not know the individual and collective effects of expression changes of constituent genes on the function of a pathway, researchers have little guidance on how to represent gene expression levels to reflect how a pathway is actually perturbed. Simply altering the expression of a fraction of constituent genes is thus questionable, given the complexity of gene expression patterns in actual biological processes and the largely unknown collective effects of different genes within a pathway.

Another strategy uses well-characterized, experimental gene expression data for a certain condition for which specific pathways (i.e., ‘hallmark pathways’) are known to be perturbed [8, 10, 11]. This approach assesses the performance of an analysis method by its ability to correctly assign high rankings to hallmark pathways from a list of significant pathways. However, because hallmark pathways typically form only a small subset of the pathways perturbed in an experimental condition, a method that identifies more or assigns higher rankings to hallmark pathways may not necessarily (even though it is quite likely to) be superior to one that identifies more non-hallmark pathways that are actually perturbed.

Yet another approach identifies perturbed pathways via the consensus of multiple analysis methods applied to the same dataset, on the assumption that the likelihood of a pathway being a true positive is higher under such a consensus [7]. This strategy, however, can yield biased conclusions because similar methods tend to generate similar results. That is, a pathway can often be identified concurrently by multiple methods, not because it is truly perturbed but because the methods considered employ similar approaches. Although reducing the weighting of methods with highly correlated results partially alleviates this issue, many datasets need to be analyzed to estimate such correlations [7].

In light of these drawbacks, an approach to assess the performance of PA methods without reference to unverified gold standards is desirable. In this report, we propose a new strategy that achieves this goal by introducing two metrics, *recall* and *discrimination*, each of which reflects a different aspect of what an effective PA method should satisfy without recourse to uncontrolled assumptions. Recall is a measure of *consistency*: an effective method should provide consistent results when applied to multiple datasets corresponding to the same experimental condition, and show increased consistency with increasing sample size. Discrimination is a measure of the difference in results obtained when a method is applied to datasets that originate from sufficiently different experimental conditions. Because the two metrics are based on comparing or contrasting PA results, calculating recall and discrimination does not require an unverified gold standard. The two metrics are mutually complementary. A trivial or highly biased method that preferentially identifies certain pathways regardless of a change in experimental condition could exhibit high recall, but by definition would also show low discrimination. In contrast, a method that identifies distinct pathways for two conditions regardless of whether they are the same or different would show high discrimination but low recall. A genuinely effective PA method would need to score sufficiently high on both metrics.

Our strategy for calculating recall is to randomly resample one large dataset consisting of many samples to form a group of smaller sub-datasets that represent the same experimental condition. To calculate discrimination, we use two large datasets from different conditions, resampling each dataset as when calculating recall. The calculation of recall is in accord with the general procedure of biomedical studies, in which a hypothesis is first generated from an experiment by using a few samples, after which it is validated in a replication that involves a large number of samples. If we use a PA method with high recall to formulate a hypothesis, we expect that it will be validated in a subsequent large-scale experiment. In addition, we expect that a highly discriminating PA method is also more likely to extract information that is specific to a particular experimental condition.

**Fig. 1:**
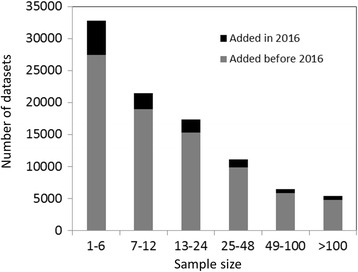
Gene Expression Omnibus (GEO) dataset sample size distribution. The majority of datasets deposited in GEO contain a small number of samples. Over 34% of datasets deposited in GEO before 2016 contain ≤6 samples, and over 57% of them contain ≤12 samples. This distribution remains mostly the same for datasets added to GEO in 2016

This resampling procedure allows us to compute recall and discrimination for sub-datasets of different sizes and to account for the effect of sample size, an issue not fully addressed in previous studies. This is important because most publicly available datasets have small sample sizes—a situation that presents an additional challenge for assessing pathway analyses. For example, a search in the Gene Expression Omnibus (GEO) database [12] revealed that 33% of expression datasets deposited before 2016 contained 6 samples or less, and that 56% contained 12 samples or less. Importantly, the latter fraction was 64% for datasets deposited in 2016 (as of October 2016; Fig. 1). The requirements for high recall and discrimination allow us to identify methods that make the best use of limited sample sizes.

To test the proposed strategy, we implemented the two metrics to assess the performance of six PA methods in identifying significantly perturbed pathways in 10 gene expression datasets. We also used 14 gene expression datasets to further evaluate our strategy. We considered three well-established methods—over-representation analysis (ORA) [5], gene set analysis (GSA) [13], and gene set enrichment analysis (GSEA) [14]—as well as a method formulated according to the general modular framework proposed in [11], which we denote as the aggregate fold change (AFC) method. By treating the two options for GSEA and AFC as separate methods, we tested a total of six methods. We systematically compared these methods and showed that recall and discrimination help reveal their strengths and weaknesses in more detail than previously observed.

## Methods

We used our proposed metrics to evaluate PA methods, with respect to a number of gene expression datasets containing multiple samples from two cohorts (treatment and control). Below, we describe the preparation of these gene expression datasets, the pathway database, the PA methods evaluated, the definitions of recall and discrimination, and the computational evaluation of these measures.

### Pathway database and gene expression data

We downloaded the Molecular Signatures Database (MsigDB) C2 collection of gene sets [14], provided by the Broad Institute, Cambridge, MA [http://software.broadinstitute.org/gsea/msigdb], in January 2016. This database contained pathway information curated from multiple online databases, including the Kyoto Encyclopedia of Genes and Genomes (KEGG), Reactome, and BioCarta. By limiting the range of gene set sizes to 20–400, we obtained a database of 892 pathways containing 8,791 human genes in total.

We selected publicly available human gene expression microarray datasets obtained under a variety of experimental conditions. Each dataset contained more than 60 samples, was extracted from multiple tissue types (blood, diseased tissues, and cell lines), and represented different types of diseases. Of the datasets meeting these criteria, we randomly selected 10 for our study (Table 1). They included eight downloaded from the GEO repository (http://www.ncbi.nlm.nih.gov/geo/) in February 2016 [studies of Alzheimer’s disease (AD) [15], Parkinson’s disease (PD) [16, 17], Crohn’s disease [18], influenza vaccination [19], breast cancer [20, 21], lung cancer [22, 23], burn injury [24], and trauma injury [25], and two datasets downloaded at the same time from the connectivity map project of the Broad Institute (https://www.broadinstitute.org/cmap/) [breast cancer cell line MCF7 and prostate cancer cell line PC3 after drug treatment [26]. We normalized the raw gene expression data, which we downloaded separately for each dataset, in the same way by using the Robust Multi-array Average method implemented in the Bioconductor R-language suite of bioinformatics tools [27].

**Table 1:** The 10 gene expression datasets used to assess the performance of pathway analysis methods

Dataset ID^a^	Study	Tissue Type	Sample Size		References
			Treatment	Control	
GSE5281	Alzheimer’s disease	Brain tissue	35	38	[15]
GSE20295	Parkinson’s disease	Brain tissue	40	53	[16, 17]
GSE3365	Crohn’s disease	Blood	59	42	[18]
GSE48018	Influenza vaccination	Blood	110	111	[19]
GSE20194	Breast cancer	Cancer tissue	222	56	[20, 21]
GSE4115	Lung cancer	Bronchial epithelium	97	90	[22, 23]
GSE37069	Burn injury	Blood	29	36	[24]
GSE36809	Trauma injury	Blood	75	37	[25]
N/A	Drug treatment of MCF7 cells	Cell line	57	323	[26]
N/A	Drug treatment of PC3 cells	Cell line	32	184	[26]

^a^Gene Expression Omnibus (GEO) database ID

In addition to the 10 datasets above, we also downloaded 14 gene expression microarray datasets from the GEO repository in August 2017 to further evaluate our strategy (Additional file 1: Table S1). These included nine relatively large datasets (sample size > 20) and five relatively small datasets (sample size < 20). The diseases represented in these datasets included AD, PD, and influenza infection, which were also represented in the original 10 datasets, and four new diseases, i.e., type I diabetes, type II diabetes, systemic lupus erythematous (SLE), and bacterial pneumonia.

### PA methods

We tested four PA methods: over-representation analysis (ORA) [5], gene set analysis (GSA) [13], gene set enrichment analysis (GSEA) [14], and the aggregate fold change (AFC) technique—a method formulated according to the general modular framework proposed in [11]. For GSEA and AFC, two options to obtain significant pathways are available: gene-label permutation and sample-label permutation. By treating these options as separate methods, we formed six variations in total: ORA, GSA, GSEA (gene-label permutation), GSEAs (sample-label permutation), AFC (gene-label permutation), and AFCs (sample-label permutation). Detailed descriptions of these methods can be found in the original literature and many reviews [11, 28]. Here, we describe them only briefly below.

ORA identifies significant pathways in two steps. It first identifies genes that are differentially expressed between two cohorts (e.g., treatment and control), as determined by a statistical test such as Student’s *t*-test, where the differential expression is considered significant if the *t*-test P value is less than a cutoff. Subsequently, it counts the number of differentially expressed genes in each pathway and tests for over-representation of these genes in all pathways, using a hypergeometric test. It considers a pathway as significant if the over-representation *P* value is less than a cutoff.

GSA, GSEA, and AFC, instead of identifying genes differentially expressed between experimental and control treatments within pathways, first compute a score for each pathway by using the expression levels of all its constituent genes. To calculate this score, GSA uses a maxmean statistic that aggregates the signed scores of individual genes, where the sign and magnitude represent the direction of regulation (up/down) and the differential expression level, respectively, of a gene. For each pathway, it computes a mean positive score and a mean negative score, and selects the score with the larger absolute value as the pathway score. GSEA uses a weighted Kolmogorov-Smirnov test to calculate the pathway score, ranking all genes by their significance and testing the distribution of each pathway’s constituent genes along the rank-ordered gene list. It then assigns a high score to a pathway whose member genes along the list are significantly clustered.

In AFC, we calculated the mean fold-change for each gene, that is, the difference between the mean log-transformed gene expression values for samples in the treatment and control cohorts, and defined the pathway score as the average mean fold-change of all genes in the pathway. We then used the pathway scores of gene expression datasets to perform null hypothesis tests and estimated each pathway’s significance by its *P* value, defined as the probability that the pathway score for a random dataset is greater than the score from the actual data. We considered a pathway as significant if its *P* value was less than a designated cutoff (i.e., 0.05). We performed the null hypothesis test by generating randomized datasets, either with permutations of gene labels or sample labels. We evaluated both approaches for GSEA and AFC. In GSA, the two permutation methods were combined into a single re-standardization procedure.

Different PA methods may identify different numbers of significant pathways for the same *P*-value cutoff. To eliminate variation in the number of significant pathways, we fixed the number for all methods. We used P values to rank-order the pathways for each method, and from each method we selected the top 20 and the top 50 most significantly up-regulated pathways, as well as the top 20 and the top 50 most significantly down-regulated pathways and used them to evaluate the performance of a method.

### Metrics for evaluating PA methods

**Fig. 2:**
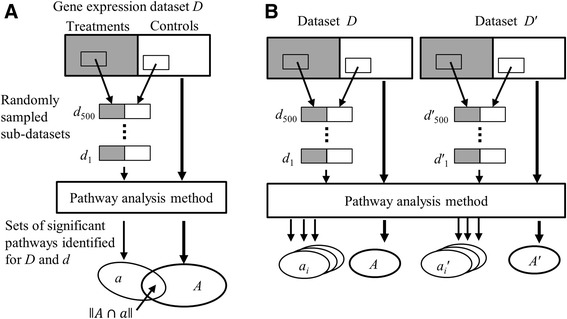
The procedure used to compute two metrics, (**a**) recall and (**b**) discrimination. Recall provides a measure of consistency of the overlap between the pathways identified from the original dataset with those obtained from sub-datasets randomly sampled from the original dataset. The computation of discrimination employs two datasets from different studies and multiple sub-datasets resampled from each of the two datasets. Discrimination measures the fraction of sub-datasets that yield higher recall with the original dataset from which they were resampled in comparison to the recall for another randomly selected dataset

Figure 2 illustrates the procedures we used to calculate recall and discrimination. We assume that a gene expression dataset *D* contains *N* samples from two cohorts (i.e., treatment and control). *D* is randomly resampled without replacement to obtain a small sub-dataset *d* of *n* samples (*n* < *N*). Because *d* is a subset of *D* and represents the same experimental condition, a PA method should ideally identify the same set of significant pathways for both datasets. Moreover, because *D* contains a larger number of samples than *d*, it should yield the most relevant set of significant pathways. We thus evaluate a PA method by applying it to datasets *D* and *d* and identifying two sets of significant pathways *A* and *a*, respectively. The recall, *r*, is then defined as

1 $$ r\left(A,a\right)=\left\Vert A\cap a\right\Vert /\left\Vert A\right\Vert $$

where ǁ*A*∩*a*ǁ represents the number of pathways contained in both sets *A* and *a*, and ǁ*A*ǁ represents the number of pathways in set *A*. Thus, *r* represents the fraction of significant pathways identified from the smaller sub-dataset among all significant pathways identified from the original dataset.

To examine the effect of sample size *n* on the performance of each of the six methods, we randomly resampled *D* without replacement to generate 500 sub-datasets of size *n* (*n* = 6, 12, 24, 48) and calculated 500 recall values by using each method for each sub-dataset. The mean and distribution of these recall values were then compared across the six methods for each sample size.

The recall *r*, as defined by Equation (1), could be biased by the sizes of sets *A* and *a*. Any pathway could be identified as significant for both *D* and *d* by chance, whose probability increases when a method identifies more pathways as significant. This leads to an increase in recall. Consequently, recall favors methods that identify a greater number of significant pathways. To reduce such a bias, we fixed the number of pathways identified by each method (e.g., only considering the top 20 or top 50 most significant pathways).

The discrimination metric *s* can be defined with respect to two datasets *D* and *D'*, each representing a different experimental condition. From *D* and *D'*, we create sub-datasets *d* and *d'*, respectively, as described above. By using a PA method and our procedure, we obtain four sets of significant pathways: *A* and *A*' from the original datasets *D* and *D*', respectively, and *a* and *a*' from the corresponding sub-datasets *d* and *d*'. Because *D* and *D'* represent different experimental conditions, *A* and *A*', as well as *a* and *a*', are expected to contain both distinct and shared pathways. The differences between *A* and *A*' and between *a* and *a*' make it possible to correctly associate *a* with *A* and *a*' with *A*'. We do this by computing recall and associating *a* with *A* if *r*(*A*, *a*) > *r*(*A*', *a*) and *a*' with *A*' if *r*(*A*', *a*') > *r*(*A*, *a*'). Correctly associating *a* and *a*' with *A* and *A′*, respectively, becomes difficult when fewer distinct pathways are identified. Hence, we define discrimination *s*, which measures the ability of a method to identify distinct pathways, as the number of sets *a* and *a*' that are correctly associated with *A* and *A*', respectively, divided by the total number of sets *a* and *a*':

2 $$ s=\left[\left\Vert {a}_i\left|r\left(A,{a}_i\right)>r\left({A}^{\hbox{'}},{a}_i\right)\right.\right\Vert +\left\Vert {a_i}^{\hbox{'}}\left|r\left({A}^{\hbox{'}},{a_i}^{\hbox{'}}\right)\right.>r\left(A,{a_i}^{\hbox{'}}\right)\right\Vert \right]/2M. $$

where pathway sets *a*_*i*_ and *a*_*i*_', with *i* = 1, 2, …, *M*, are computed for *M* randomly resampled sub-datasets *d*_*i*_ and *d*_*i*_', respectively.

Discrimination is close to 1 when a method identifies more distinct significant pathways for datasets representing different experimental conditions. Conversely, if sets *A* and *A*' or *a* and *a*' are nearly identical, discrimination is close to zero. The concept of discrimination can also be extended to more than two datasets, each representing a different condition: *r*(*A*, *a*_*i*_) should be greater than any other recall value *r*(*B*, *a*_*i*_), where *B* is a set of significant pathways for datasets other than *D*.

Although discrimination depends on recall, the two metrics are complementary: discrimination considers only those pathways that are specific to particular experimental conditions and does not directly reflect the overall extent to which all significant pathways are sufficiently represented in each condition. In contrast, recall considers all significant pathways, both specific and non-specific. A trivial method that yields the same result for any dataset would show high recall but low discrimination. Taken together, recall and discrimination can provide a balanced and objective evaluation of PA methods.

## Results

### Recall of PA methods

**Fig. 3:**
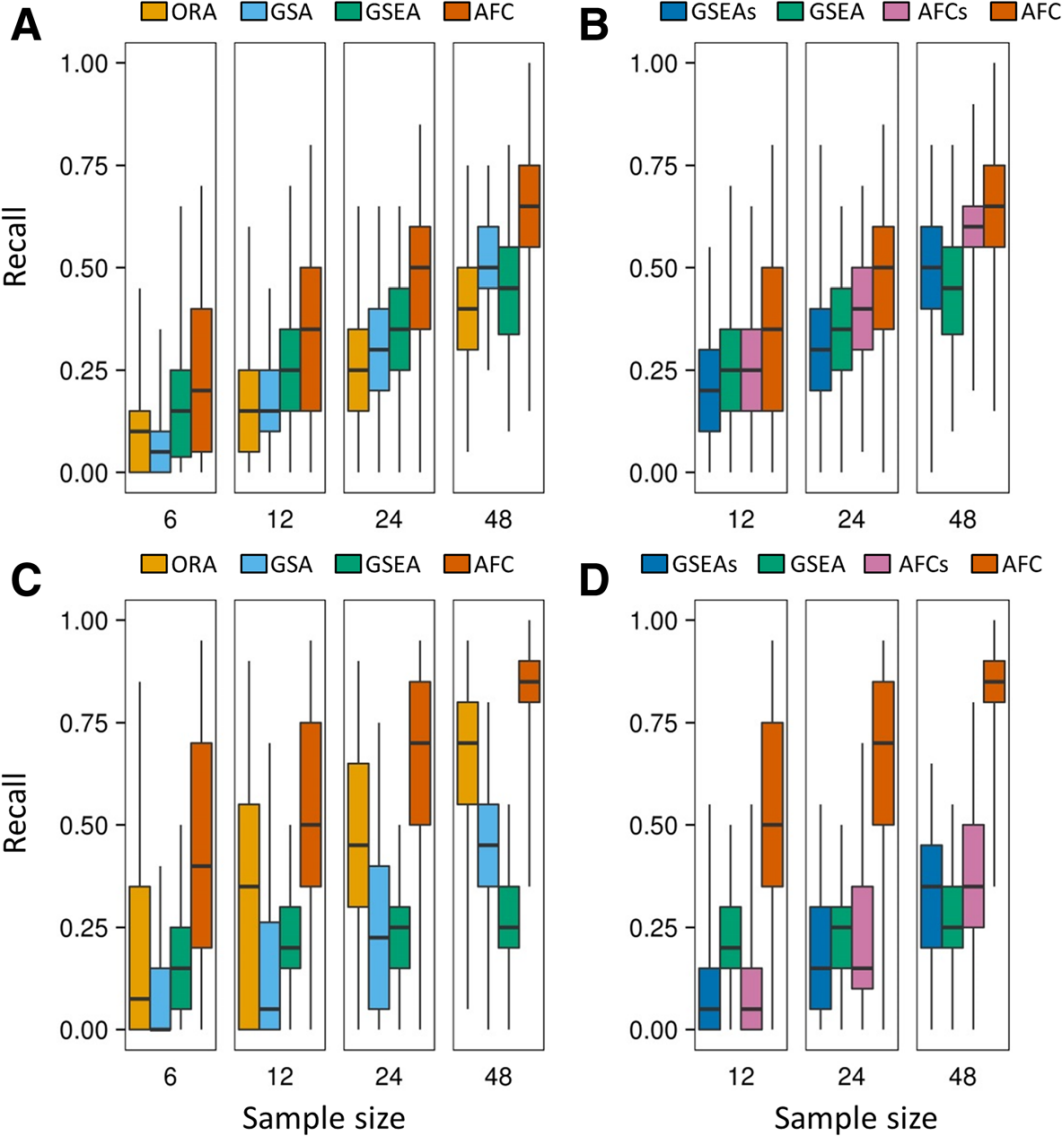
Comparison of recall values computed for the six pathway analysis methods, based on the top 20 most significantly up-regulated (**a**, **b**) and top 20 most significantly down-regulated pathways (**c**, **d**), which each method identified for the Parkinson’s disease dataset. For each box-and-whisker plot, the horizontal line, top and bottom sides of the box, and vertical line show the median, upper and lower quartiles, and range, respectively, of the recall values. The comparisons are separated into two groups for clarity and to distinguish the two different permutation methods. **a** and **c** compare methods ORA, GSA, GSEA, and AFC. **b** and **d** compare methods GSEAs, GSEA, AFCs, and AFC. The sample size corresponds to the number of samples contained in the sub-datasets randomly resampled from the original datasets

We computed recall for each of the six PA methods that were applied to each of the 10 gene expression datasets, and evaluated their performance in identifying a fixed number (20 or 50) of significantly up- or down-regulated pathways for resampled sub-datasets of different sizes (6, 12, 24, and 48). Figure 3 shows the recall distributions for the resampled PD datasets, illustrating the top 20 most significantly up- or down-regulated pathways for each method. The results for each of the 10 datasets, where each method identified the top 20 or the top 50 significant pathways, can be found in Additional file 2: Figures S1–S10. For clarity, we classified the methods into two groups (group 1: ORA, GSA, GSEA, and AFC; group 2: GSEAs, GSEA, AFCs, and AFC). The first group compared four different methods, using gene-label permutation in testing the null hypothesis. The second group compared GSEA and AFC, using gene-label (GSEA, AFC) and sample-label (GSEAs, AFCs) permutations. We also computed the average recall as a measure of the overall performance of each method for the 10 datasets (Table 2). We did not use resampled sub-datasets of size 6 in comparing the second group of methods because the small sample size precluded a sufficient number of sample-label permutations (only 10 distinct permutations for a dataset of 3 treatment and 3 control samples).

**Table 2:** Average recall values for the six pathway analysis (PA) methods, using the 10 gene expression datasets

Sample Size	ORA	GSA	GSEA	GSEAs	AFC	AFCs
Significantly up-regulated pathways						
20 MSP^a^						
6	0.19	0.13 ^b^	0.25		**0.44** ^**c**^	
12	0.28	0.31	0.30	0.23	**0.54**	0.31
24	0.38	0.45	0.34	0.35	**0.64**	0.45
48	0.51	0.58	0.40	0.48	**0.74**	0.60
50 MSP						
6	0.23	0.27	0.39		**0.50**	
12	0.33	0.43	0.46	0.35	**0.59**	0.42
24	0.42	0.55	0.52	0.46	**0.68**	0.55
48	0.55	0.68	0.60	0.59	**0.78**	0.68
Significantly down-regulated pathways						
20 MSP						
6	0.37	0.14	0.35		**0.56**	
12	0.49	0.31	0.40	0.27	**0.64**	0.34
24	0.58	0.43	0.45	0.38	**0.72**	0.46
48	0.68	0.55	0.49	0.51	**0.80**	0.60
50 MSP						
6	0.39	0.29	0.48		**0.59**	
12	0.51	0.45	0.54	0.41	**0.66**	0.47
24	0.60	0.56	0.60	0.51	**0.73**	0.59
48	0.70	0.68	0.65	0.63	**0.81**	0.70

^a^MSP most significant pathway

^b^Underlined text indicates the minimal value in a row

^c^Bold text indicates the maximal value in a row

The computations are based on each method’s identification of the top 20 or 50 most significantly up- or down-regulated pathways for all datasets (and 500 randomly resampled sub-datasets for each original dataset). The sample size corresponds to the number of samples contained in the sub-datasets. A higher average recall reflects a more consistent PA method

Figure 3 (and Additional file 2: Figures S1–S10) shows that the recall of a method generally decreased as the sub-dataset sample size decreased—a trend observed for all ten datasets and different numbers of significantly up- or down-regulated pathways. This observation reflects the challenge in analyzing small datasets encountered with any method. Compared with other methods, AFC almost always produced the highest recall, whereas the other three methods (ORA, GSA, and GSEA) often produced low recall (Fig. 3a and c). Comparisons between GSEAs and GSEA, and between AFCs and AFC, showed that sample-label permutation often yielded lower recall than did gene-label permutation (Fig. 3b and d). The average recall values in Table 2 corroborated these findings. The table shows the highest average recall values (in bold) for AFC in identifying different numbers of significant pathways for sub-datasets of different size. GSEAs and AFCs showed average recall values smaller than those of their counterparts, GSEA, and AFC. Although the comparisons of other methods were inconclusive, ORA consistently produced the lowest average recall (underlined in Table 2) in identifying the top 50 most up-regulated pathways, whereas GSEAs often produced the lowest average recall in identifying the top 50 most down-regulated pathways. We note that recall for GSEA did not considerably increase with sample size in the analysis of some datasets, for example, the AD dataset (Additional file 2: Figure S1, plots C, D, G, and H) and the burn injury dataset (Additional file 2: Figure S7, plots A, B, E, and F). For these cases, the selection of sample-label permutation (i.e., GSEAs) considerably improved recall, especially for datasets with a large number of samples.

### Discrimination of identified pathways

**Fig. 4:**
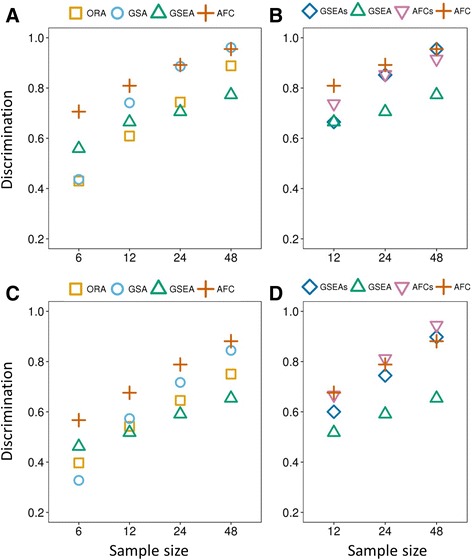
Comparison of discrimination values computed for the six pathway analysis methods, based on the top 20 most significantly up-regulated (**a**, **b**) and top 20 most significantly down-regulated pathways (**c**, **d**), which each method identified for the 10 gene expression datasets. The sample size corresponds to the number of samples contained in the sub-datasets randomly resampled from the 10 datasets

We computed the discrimination values of significantly up- or down-regulated pathways identified by the six PA methods. Figure 4 shows the comparative results, where the top 20 (and the top 50) most significantly up- or down-regulated pathways were identified by each method for sub-datasets of different sizes. Similar to recall, discrimination also generally decreased with decreasing sample size, highlighting the greater difficulty of identifying pathways specific to experimental conditions with fewer samples. AFC performed better than all other methods when applied to datasets with smaller sample sizes (6 and 12); discrimination was 19% higher than that of the second best method (GSEA) and 30% higher than that of ORA or GSA for datasets of 6 samples. The difference in discrimination between AFC and other methods decreased (or vanished) for datasets with larger sample sizes (24 and 48). Nevertheless, AFC still outperformed other methods, except for one case where GSA outperformed AFC. The comparison between GSEAs and GSEA suggests that using sample-label permutation instead of gene-label permutation considerably improves discrimination and that the improvement is more pronounced for larger sample sizes. However, we did not observe a significant difference between AFCs and AFC.

### Reproducibility of recall

We used 9 additional large datasets (Additional file 1: Table S1) to test whether the relative performance of the six methods assessed with the first 10 datasets would still hold for other conditions. The results in all 9 datasets (Additional file 2: Figures S11–S19) corroborated the original findings: recall decreased as the sub-dataset sample size decreased; AFC almost always showed the highest recall; and sample-label permutation (GSEAs and AFCs) often showed lower recall than did gene-label permutation (GSEA and AFC). As expected, the trends for different datasets of the same disease were similar. For example, recall for GSEA did not considerably increase with sample size for the top 20 most significantly down-regulated pathways in the three AD datasets (Additional file 2: Figures S1, S11, and S12, plots C and D). Importantly, the six methods’ rank order in recall was almost identical for the three AD datasets and the three PD datasets (Additional file 2: Figures S2, S13, and S14).

### Comparison of small and large datasets of the same disease

In our initial analysis, we resampled an original large dataset to form small sub-datasets in order to investigate the effect of sample size. Because sample sizes for most gene expression datasets in public databases, such as GEO, are small, we further tested the ability of our strategy to assess PA methods by directly using small datasets of the same disease. We employed the six PA methods to predict significant pathways for five small datasets (sample size < 20) and compared the results with those for five large datasets of the same disease (sample size > 20, Additional file 1: Table S1). We computed the proportion of overlapping pathways of the top 50 significant pathways separately identified for the small and large datasets, which is equivalent to the recall computed by re-sampling a large dataset. Therefore, we superimposed these proportions onto the box-and-whisker plots of recall for the large datasets (Additional file 2: Figures S11–S14, S17). For most comparisons, this proportion was much smaller than the corresponding recall for a large number of resampled sub-datasets (Additional file 2: Figures S20–22). This is not unexpected because potential differences in studies (e.g., experimental design, technical procedure) are accentuated for small sample sizes. Such effects could be study- or disease-specific, which may have caused the very low proportions for the two small PD datasets regardless of PA method (Additional file 2: Figure S21). For the two small AD datasets (Additional file 2: Figure S20) and one small influenza infection dataset (Additional file 2: Figure S22), this proportion and recall showed similar trends. The ranks of the six methods ordered by this proportion were generally similar to those ordered by recall.

## Discussion

In this work, we proposed a novel strategy for evaluating PA methods without reference to gold standards. The strategy consists of two mutually complementary metrics, recall and discrimination, and a resampling procedure that generates multiple small sub-datasets of a specific size from two or more large datasets. These metrics address two different desired aspects of a high-performing PA method. In typical biomedical data-driven inferences, we aim to formulate hypotheses by analyzing samples of limited size, and attempt to replicate the initial analysis by using additional independent samples. By calculating recall, we can critically assess the performance of a method in this context in terms of its consistency in identifying significantly perturbed pathways in randomly resampled small sub-datasets. The higher the recall, the more likely it is that pathways identified from small datasets will also be found in larger datasets. Typically, we also expect to find distinct pathways reflecting specific experimental conditions in different studies—a feature not necessarily shared by analysis tools that exhibit only high recall. By calculating discrimination, we can measure the extent to which a PA tool yields different pathways from two unrelated datasets.

We demonstrated our proposed strategy by assessing the performance of six PA methods. Because there is no verified gold standard to directly assess the exact performance of PA methods, we assessed our strategy by *1*) analyzing changes in recall and discrimination for multiple datasets comprising different conditions, *2*) comparing our pathway performance evaluation results with previous comparative studies of PA methods, and *3)* comparing overlapping significant pathways predicted for a pair of datasets of the same disease.

Our performance comparisons for the six PA methods highlighted the common challenge in reliably identifying significant pathways from small datasets. Decreasing the sample size steadily decreased both recall and discrimination. This trend was common for all six methods across all 10 analyzed datasets, with only a few exceptions observed for GSEA (but not GSEAs). Although each method attained different recall values for different datasets (Additional file 2: Figures S1–S10), which presumably reflects the disparate biological processes represented in each dataset, ranking the six methods by recall across the 10 datasets yielded similar results: AFC was always ranked highest, whereas ORA and GSA tied for a low rank for small datasets (size 6 or 12) and GSEA (or GSEAs) fell to a low rank for large datasets (size 48), as seen in Fig. 3 and Table 2. The consistency of performance rankings for the 10 datasets representing different experimental conditions suggests that our proposed metrics can potentially assess the inherent capability of different PA methods, with a limited number of datasets.

The recall- and discrimination-based rankings of the six PA methods, and in particular the low ranking of ORA, can be explained by the simplified assumptions adopted by the methods. ORA only uses genes deemed statistically significant by an arbitrary cutoff to identify perturbed pathways. From a systems biology perspective, a pathway may be significantly perturbed by multiple genes with statistically insignificant (marginal) changes in expression. By only counting significant genes, ORA risks neglecting the collective effects of many marginally associated genes. Sample sizes that are inadequate for effective application of Student’s t-test in determining significant genes may further deteriorate the performance of ORA in analyzing small datasets. In contrast, AFC (AFCs), GSEA (GSEAs), and GSA use measurements for all genes that show differential expression in a pathway. Consistent with this view, the recall- and discrimination-based evaluation indicated that ORA performed worse than AFC (and AFCs) in all comparisons, worse than GSEA in six out of eight comparisons of recall and three out of four comparisons of discrimination with respect to small datasets (size 6 and 12), and worse than GSA in seven out of eight comparisons of discrimination. For the remaining comparisons, the performance of ORA was comparable to that of GSA. Comparisons of ORA and GSEA in analyzing large datasets (size 24 and 48) were inconclusive.

Neither GSEA nor GSA consistently outperformed ORA in any recall- and discrimination-based comparison. This suggests potential issues with the GSEA and GSA methods. For GSEA, one problem may be the weighted Kolmogorov-Smirnov test, which has been criticized as lacking sensitivity [29]. As for GSA, we speculate that its maxmean statistic, which presumably reflects the effects of either up- or down-regulated genes on a given pathway, does not consider the possibility of a pathway being affected by both up- and down-regulated genes simultaneously. The inability to account for the effects of all genes may thus impair the performance of GSA, especially for small datasets. Our strategy arguably assessed the two methods more appropriately than a previous comparative study that ranked both GSEA and GRA lower than ORA [8].

Our strategy clearly showed differences between the two null hypothesis tests employed in AFC and GSEA, sample-label permutation and gene-label permutation, which have been extensively discussed [30, 31]. Sample-label permutation is believed to be able to reduce the false positive rate by eliminating the effects of co-expressed genes in a pathway. It is thus superior to gene-label permutation in identifying pathways that are more specifically associated with given experimental conditions [31]. However, sample size is a major constraint in applying sample-label permutations. This constraint was reflected in the substantial recall reduction for AFCs and GSEAs (relative to AFC and GSEA) with decreasing sample size (Table 2). For a sample size of 48, in particular, the recall reduction was 13–29% for AFCs (relative to AFC) and no more than 3% for GSEAs (relative to GSEA), whereas for a sample size of 12, the recall reduction increased to 29–42% for AFCs and 23–33% for GSEAs. Discrimination clearly showed the advantage of using sample-label permutation for GSEA, especially for large datasets. With sample sizes of 24 and 48, the discrimination improvement by using sample-label permutation rather than gene-label permutation was greater than 20% (Fig. 4b and d). This notable improvement is consistent with previous studies by the authors of GSEA and the strong recommendations given by independent researchers to adopt GSEA with sample-labeled permutation (i.e., GSEAs) [14, 31]. We did not observe a similar improvement for AFCs, presumably because the performance level of AFC was already high.

Our comparisons of the proportion of significant pathways that one method predicted for a small dataset and those it predicted for a large dataset of the same disease showed that for some diseases (e.g., AD and influenza infection), the ranks of the six PA methods ordered by this proportion were nearly identical to those ordered by recall (Additional file 2: Figures S20, S22). This suggests that our strategy is able to determine methods that are suitable in analyzing datasets from certain small studies and providing results that are likely to be verified in a separate large study. However, the variability of small samples may also preclude any method from producing reproducible results, as we observed for small PD datasets (Additional file 2: Figure S21). In such cases, our evaluation strategy offers little guidance.

Our study focused on developing a strategy to evaluate PA methods. We evaluated the performance of six PA methods to demonstrate our strategy. Their relative performance on additional 9 large datasets was largely consistent. However, the performance of PA methods could be data-dependent. It is possible for a method to achieve better performance on one dataset but worse performance on another. By design, our strategy provides the opportunity to evaluate a method on a large dataset from any study where the perturbed pathways are unknown.

Our strategy requires relatively large datasets to generate multiple sub-datasets through re-sampling. This could be a limitation when users need a method for a particular study that involves only small datasets. In addition, users should be cautious in choosing large datasets from studies where covariates are of concern. In such cases, the distribution of covariates (e.g., sex ratio) in the original dataset needs to be preserved in the sub-datasets. In general, this is achieved by generating a large enough number of re-sampled sub-datasets. In this case, the majority of the sub-datasets will then retain the same distribution of covariates as the original dataset. However, this may not be achieved when the size of a sub-dataset is too small or the incidence of a covariate in the original dataset is extremely low.

We used microarray data in our demonstration because, while enormous amounts of such data have been accumulated, the data are considered noisy and thus present challenges for PA methods to produce meaningful results. We emphasize that our strategy can also use any other bio-molecular data suitable for pathway analysis (e.g., RNAseq data) to evaluate PA methods.

In summary, we have demonstrated a newly developed dual-metric strategy that evaluates the performance of PA methods. Our strategy allows us to employ any number of datasets for various conditions because we need not know the truly perturbed pathways associated with each condition. In addition, it is applicable not only to PA methods that do not take into account interactions between genes, but also to more advanced methods that consider such topological information [6].

## Additional files

Additional file 1: Table S1. The 14 additional gene expression datasets used to further assess the performance of pathway analysis methods. (DOCX 41 kb)

Additional file 2: Figure S1–S22. Additional comparison of recall values computed for the six pathway analysis methods using different datasets. (PPTX 6592 kb)

## Abbreviations

AFC: Aggregate fold change
AFCs: AFC with sample-label permutation
GEO: Gene Expression Omnibus
GSA: Gene set analysis
GSEA: Gene Set Enrichment Analysis
GSEAs: GSEA with sample-label permutation
KEGG: Kyoto Encyclopedia of Genes and Genomes
MsigDB: Molecular Signatures Database
ORA: Over-representation analysis
PA method: Pathway analysis method
