# Supplementary material for: A strategy for evaluating pathway analysis methods
Source: BMC Bioinformatics. 2017 Oct 13;18:453. doi: 10.1186/s12859-017-1866-7 (PMC5640951; doi:10.1186/s12859-017-1866-7)
Supplement: Supplementary file 1 — The 14 additional gene expression datasets used to further assess the performance of pathway analysis methods. (DOCX 41 kb) [file 12859_2017_1866_MOESM1_ESM.docx]

**Table S1.** The 14 additional gene expression datasets used to further assess the performance of pathway analysis methods

| **ID** | **GEO Accession** | **Study** | **Tissue Type** | **Sample Size** | | | **Release Year** |
| --- | --- | --- | --- | --- | --- | --- | --- |
|  |  |  |  | **Treatment** | | **Control** |  |
| ***Large datasets (sample size > 20)*** | | | | | | | |
| AD36980 | GSE36980 | Alzheimer’s disease | Brain | | 32 | 47 | 2013 |
| AD53890 | GSE53890 | Alzheimer’s disease | Brain | | 24 | 17 | 2014 |
| PD8387 | GSE8397 | Parkinson’s disease | Brian | | 29 | 18 | 2008 |
| PD7621 | GSE7621 | Parkinson’s disease | Brain | | 16 | 9 | 2007 |
| T1D9006 | GSE9006 | Type I diabetes | PBMC^a^ | | 43 | 24 | 2007 |
| T2D9006 | GSE9006 | Type II diabetes | PBMC | | 12 | 24 | 2007 |
| FLU20346 | GSE20346 | Influenza infection | Blood | | 12 | 18 | 2011 |
| PNE20346 | GSE20346 | Bacterial pneumonia | Blood | | 16 | 18 | 2011 |
| SLE8650 | GSE8650 | Systemic lupus erythematous | Blood | | 38 | 21 | 2007 |
| ***Small datasets (sample size < 20)*** | | | | | | | |
| AD20146M | GSE28146 | Moderate Alzheimer’s disease | Brain | | 8 | 8 | 2011 |
| AD28146S | GSE28146 | Severe Alzheimer’s disease | Brain | | 7 | 8 | 2011 |
| AD19587DM | GSE19587 | Parkinson’s disease | Brain (Dorsal motor nucleus) | | 6 | 5 | 2010 |
| AD19587OL | GSE19587 | Parkinson’s disease | Brain (Inferior Olivary nucleus) | | 6 | 5 | 2010 |
| FLU27131 | GSE27131 | Influenza | Blood | | 7 | 7 | 2011 |

^a^Peripheral blood mononuclear cells
