# Supplementary material for: A strategy for evaluating pathway analysis methods
Source: BMC Bioinformatics. 2017 Oct 13;18:453. doi: 10.1186/s12859-017-1866-7 (PMC5640951; doi:10.1186/s12859-017-1866-7)
Supplement: Supplementary file 2 — Additional comparison of recall values computed for the six pathway analysis methods using different datasets. (PPTX 6592 kb) [file 12859_2017_1866_MOESM2_ESM.pptx]

## Slide 1
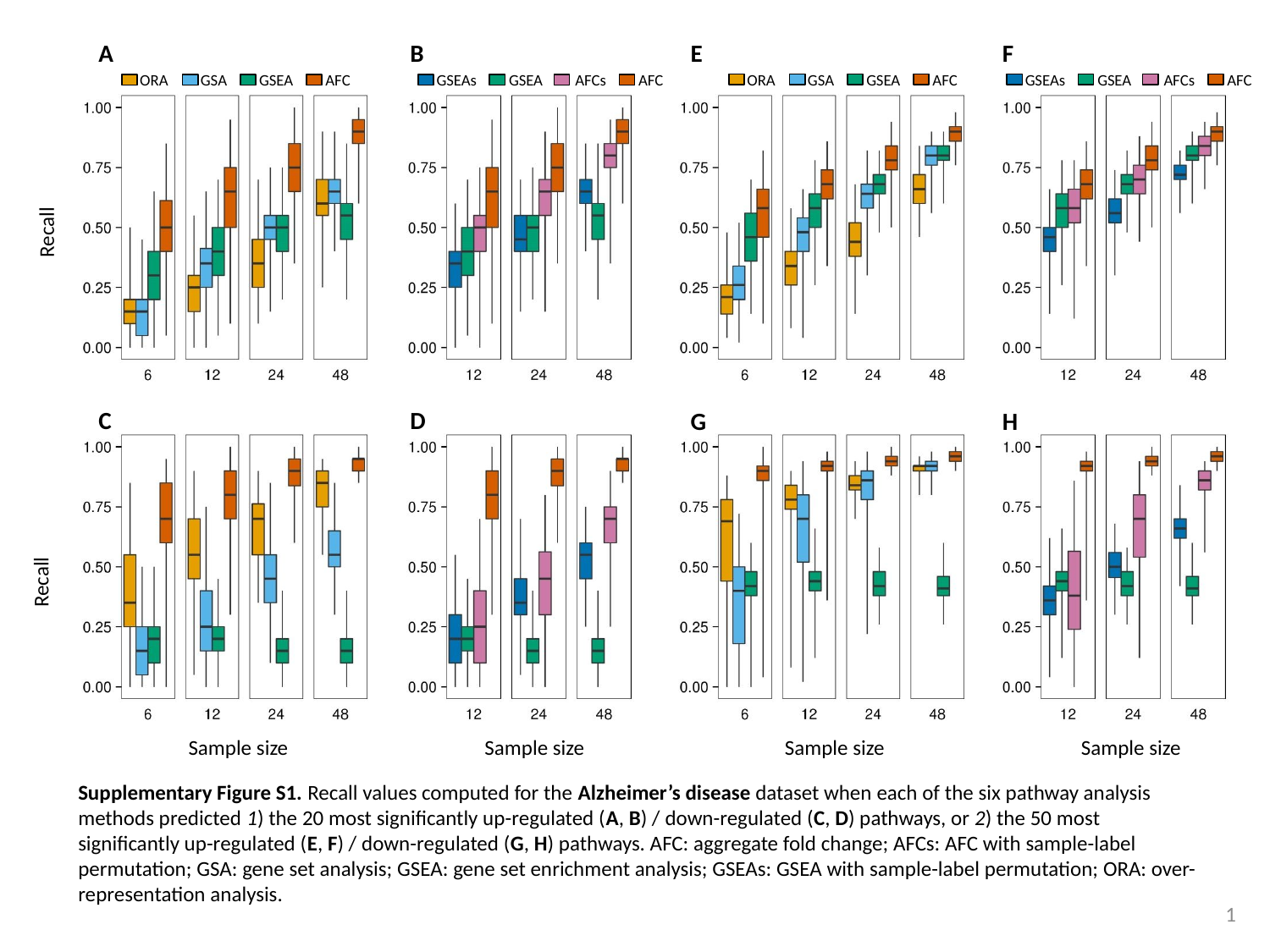

A
B
E
F
ORA GSA GSEA AFC
GSEAs GSEA AFCs AFC
ORA GSA GSEA AFC
GSEAs GSEA AFCs AFC
Recall
C
D
G
H
Recall
Sample size
Sample size
Sample size
Sample size
Supplementary Figure S1. Recall values computed for the Alzheimer’s disease dataset when each of the six pathway analysis methods predicted 1) the 20 most significantly up-regulated (A, B) / down-regulated (C, D) pathways, or 2) the 50 most significantly up-regulated (E, F) / down-regulated (G, H) pathways. AFC: aggregate fold change; AFCs: AFC with sample-label permutation; GSA: gene set analysis; GSEA: gene set enrichment analysis; GSEAs: GSEA with sample-label permutation; ORA: over-representation analysis.
1

## Slide 2
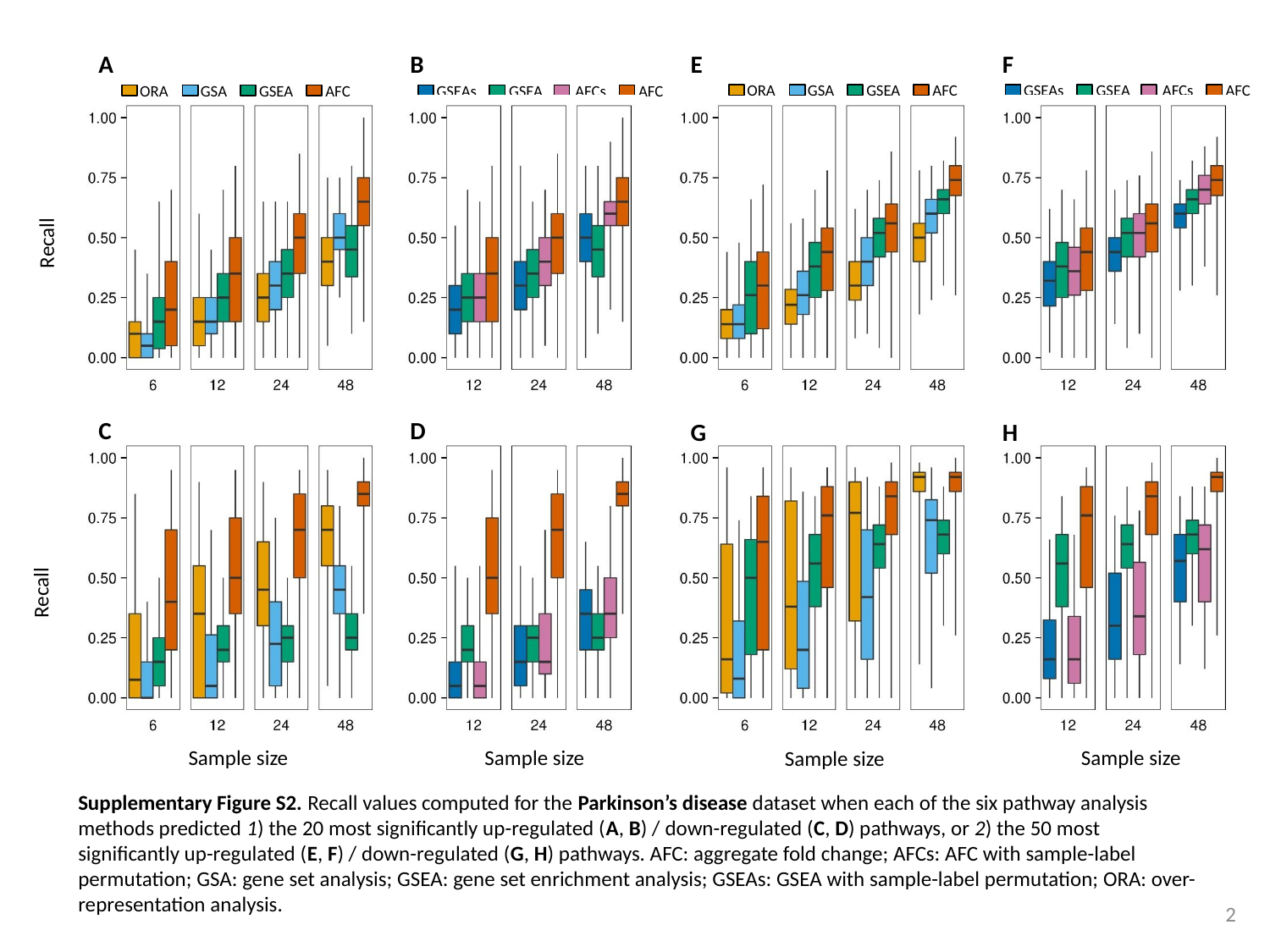

A
B
E
F
ORA GSA GSEA AFC
GSEAs GSEA AFCs AFC
ORA GSA GSEA AFC
GSEAs GSEA AFCs AFC
Recall
C
D
G
H
Recall
Sample size
Sample size
Sample size
Sample size
Supplementary Figure S2. Recall values computed for the Parkinson’s disease dataset when each of the six pathway analysis methods predicted 1) the 20 most significantly up-regulated (A, B) / down-regulated (C, D) pathways, or 2) the 50 most significantly up-regulated (E, F) / down-regulated (G, H) pathways. AFC: aggregate fold change; AFCs: AFC with sample-label permutation; GSA: gene set analysis; GSEA: gene set enrichment analysis; GSEAs: GSEA with sample-label permutation; ORA: over-representation analysis.
2

## Slide 3
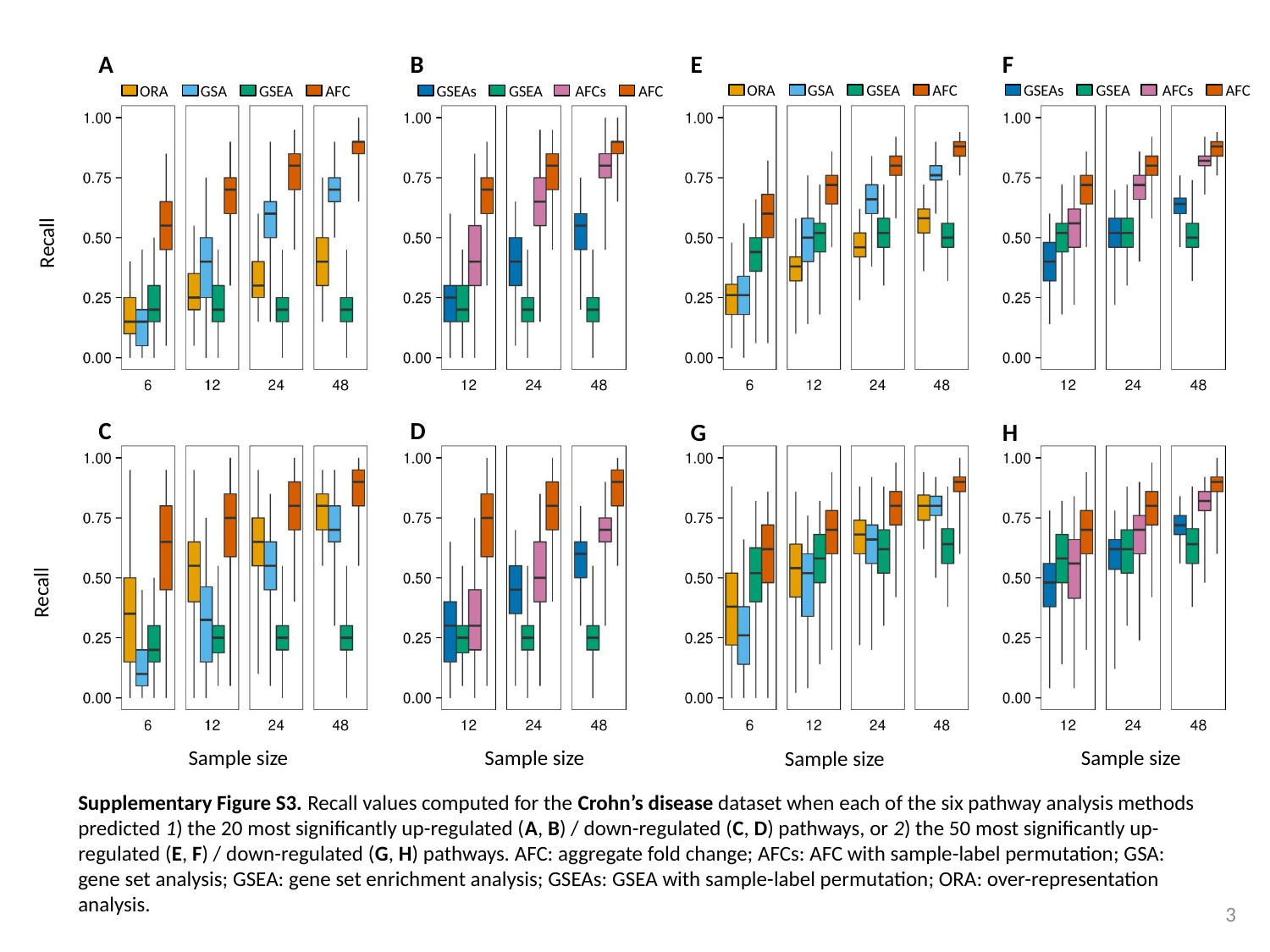

A
B
E
F
ORA GSA GSEA AFC
GSEAs GSEA AFCs AFC
ORA GSA GSEA AFC
GSEAs GSEA AFCs AFC
Recall
C
D
G
H
Recall
Sample size
Sample size
Sample size
Sample size
Supplementary Figure S3. Recall values computed for the Crohn’s disease dataset when each of the six pathway analysis methods predicted 1) the 20 most significantly up-regulated (A, B) / down-regulated (C, D) pathways, or 2) the 50 most significantly up-regulated (E, F) / down-regulated (G, H) pathways. AFC: aggregate fold change; AFCs: AFC with sample-label permutation; GSA: gene set analysis; GSEA: gene set enrichment analysis; GSEAs: GSEA with sample-label permutation; ORA: over-representation analysis.
3

## Slide 4
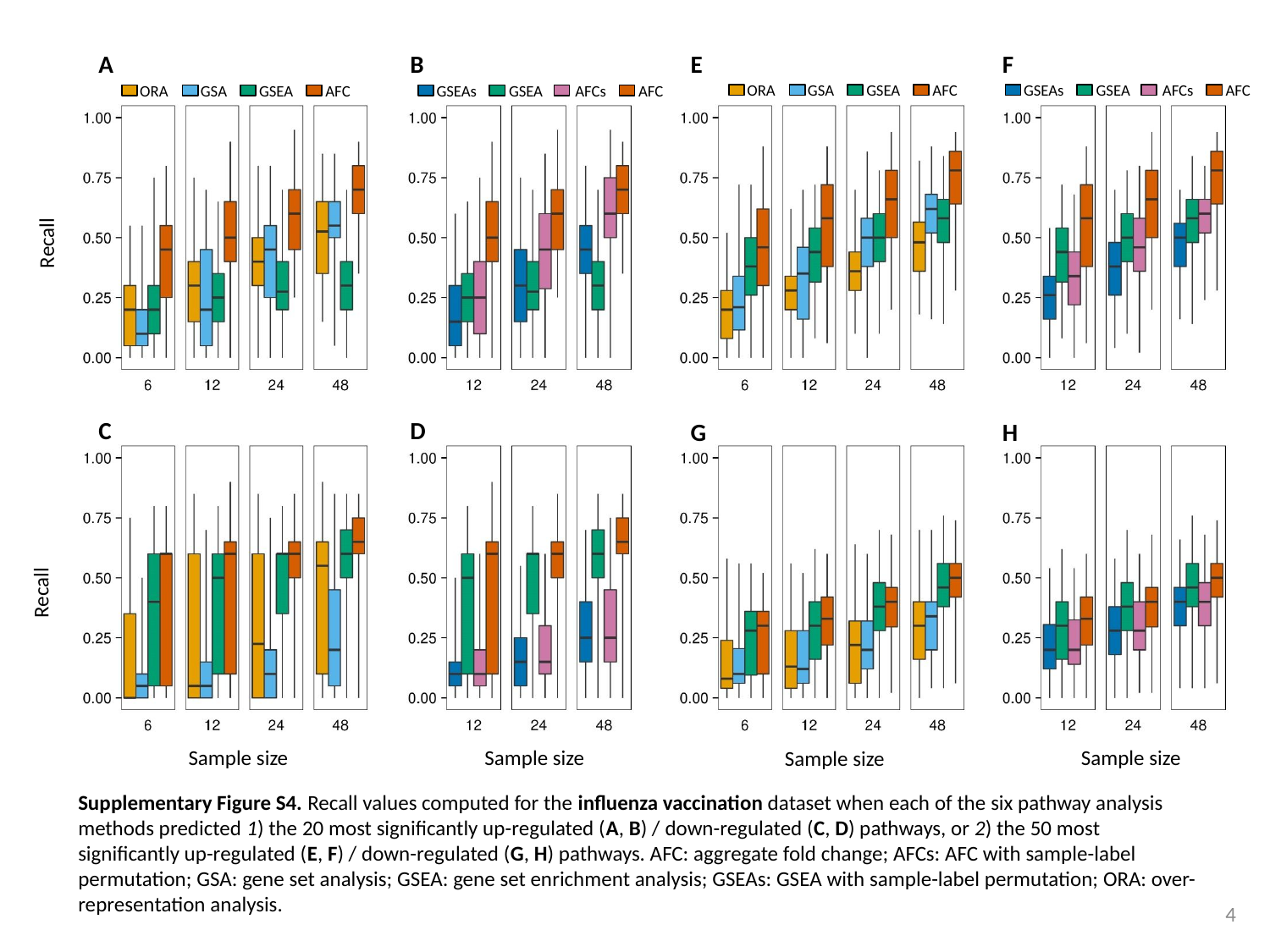

A
B
E
F
ORA GSA GSEA AFC
GSEAs GSEA AFCs AFC
ORA GSA GSEA AFC
GSEAs GSEA AFCs AFC
Recall
C
D
G
H
Recall
Sample size
Sample size
Sample size
Sample size
Supplementary Figure S4. Recall values computed for the influenza vaccination dataset when each of the six pathway analysis methods predicted 1) the 20 most significantly up-regulated (A, B) / down-regulated (C, D) pathways, or 2) the 50 most significantly up-regulated (E, F) / down-regulated (G, H) pathways. AFC: aggregate fold change; AFCs: AFC with sample-label permutation; GSA: gene set analysis; GSEA: gene set enrichment analysis; GSEAs: GSEA with sample-label permutation; ORA: over-representation analysis.
4

## Slide 5
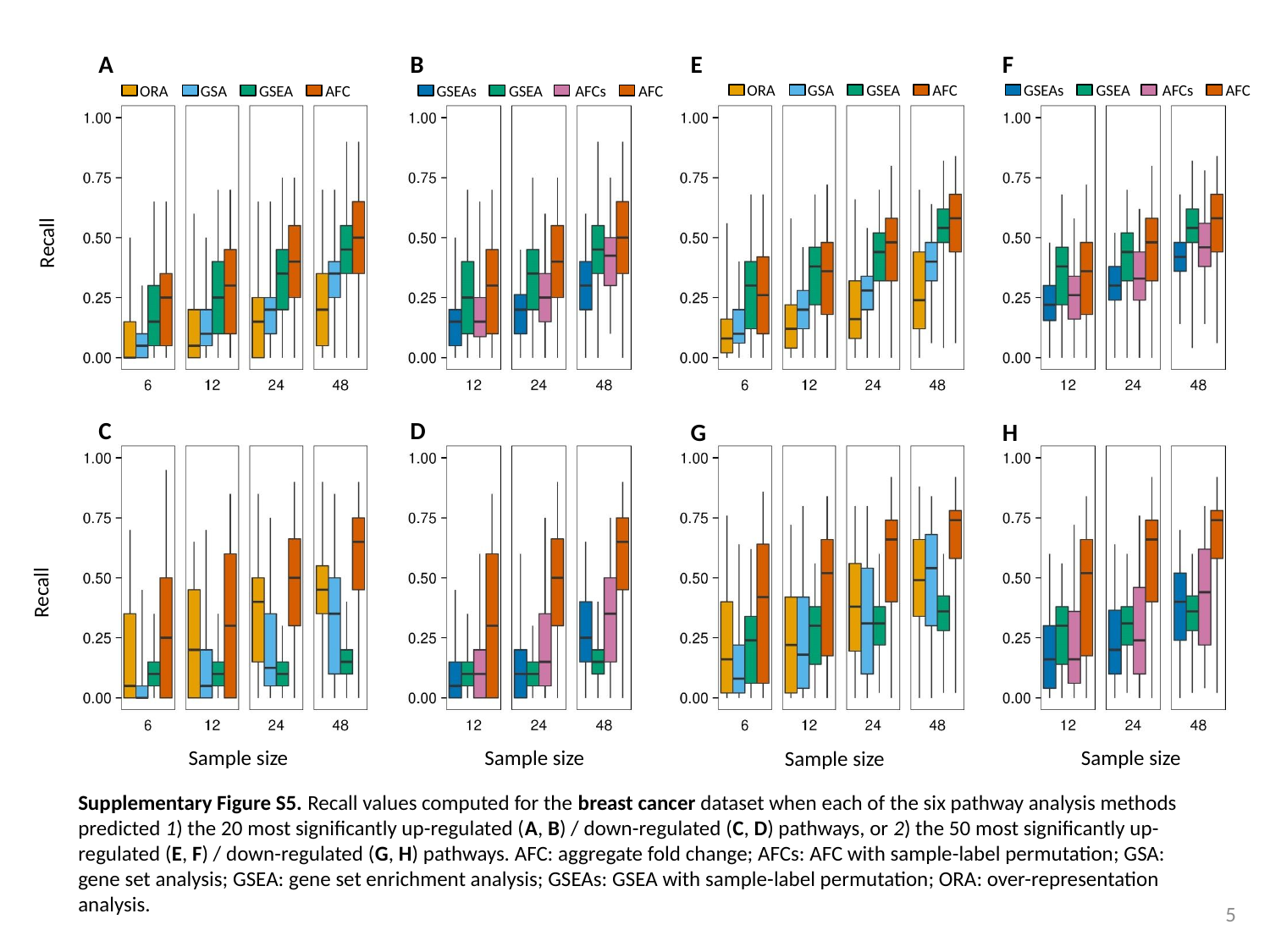

A
B
E
F
ORA GSA GSEA AFC
GSEAs GSEA AFCs AFC
ORA GSA GSEA AFC
GSEAs GSEA AFCs AFC
Recall
C
D
G
H
Recall
Sample size
Sample size
Sample size
Sample size
Supplementary Figure S5. Recall values computed for the breast cancer dataset when each of the six pathway analysis methods predicted 1) the 20 most significantly up-regulated (A, B) / down-regulated (C, D) pathways, or 2) the 50 most significantly up-regulated (E, F) / down-regulated (G, H) pathways. AFC: aggregate fold change; AFCs: AFC with sample-label permutation; GSA: gene set analysis; GSEA: gene set enrichment analysis; GSEAs: GSEA with sample-label permutation; ORA: over-representation analysis.
5

## Slide 6
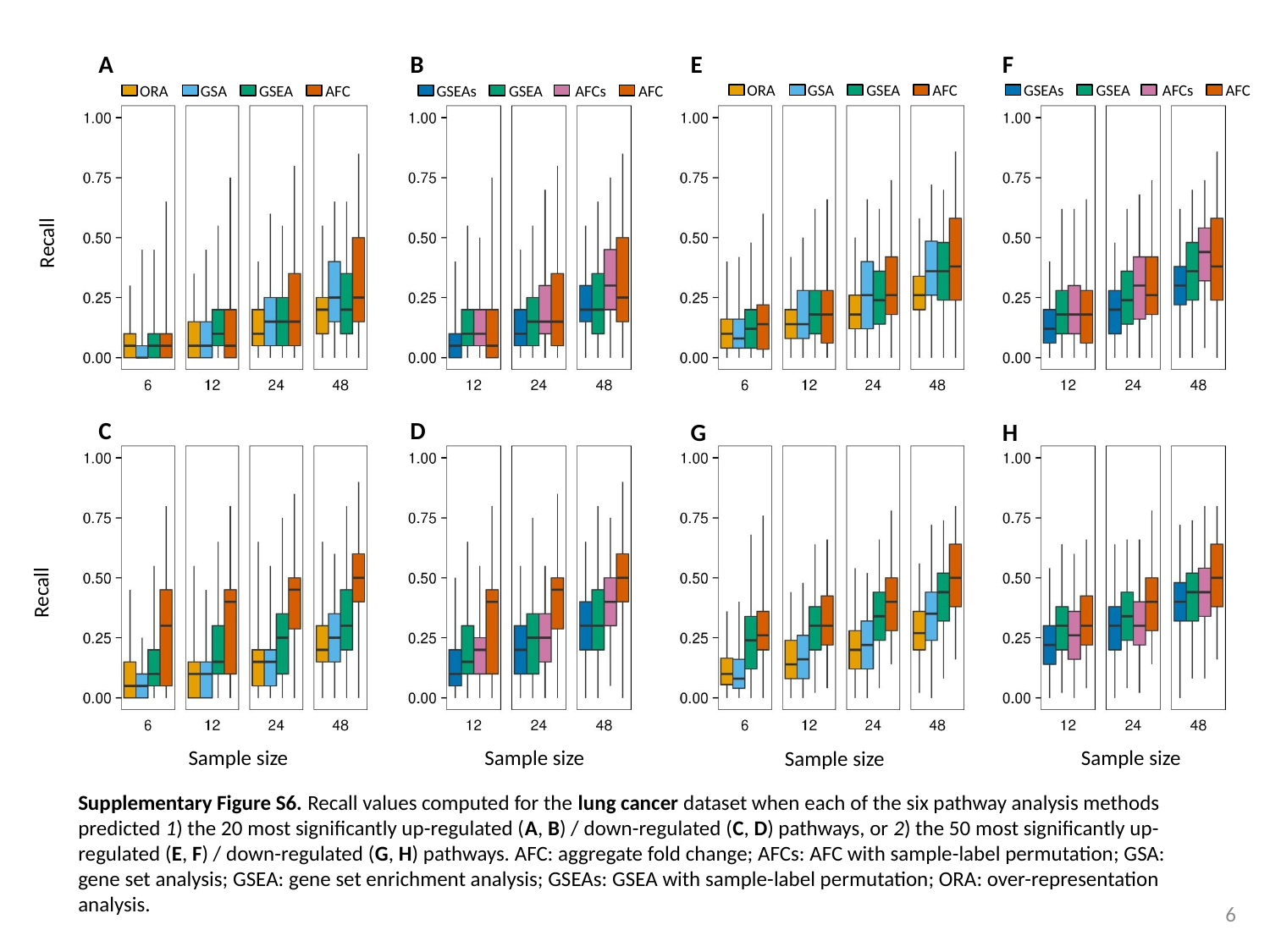

A
B
E
F
ORA GSA GSEA AFC
GSEAs GSEA AFCs AFC
ORA GSA GSEA AFC
GSEAs GSEA AFCs AFC
Recall
C
D
G
H
Recall
Sample size
Sample size
Sample size
Sample size
Supplementary Figure S6. Recall values computed for the lung cancer dataset when each of the six pathway analysis methods predicted 1) the 20 most significantly up-regulated (A, B) / down-regulated (C, D) pathways, or 2) the 50 most significantly up-regulated (E, F) / down-regulated (G, H) pathways. AFC: aggregate fold change; AFCs: AFC with sample-label permutation; GSA: gene set analysis; GSEA: gene set enrichment analysis; GSEAs: GSEA with sample-label permutation; ORA: over-representation analysis.
6

## Slide 7
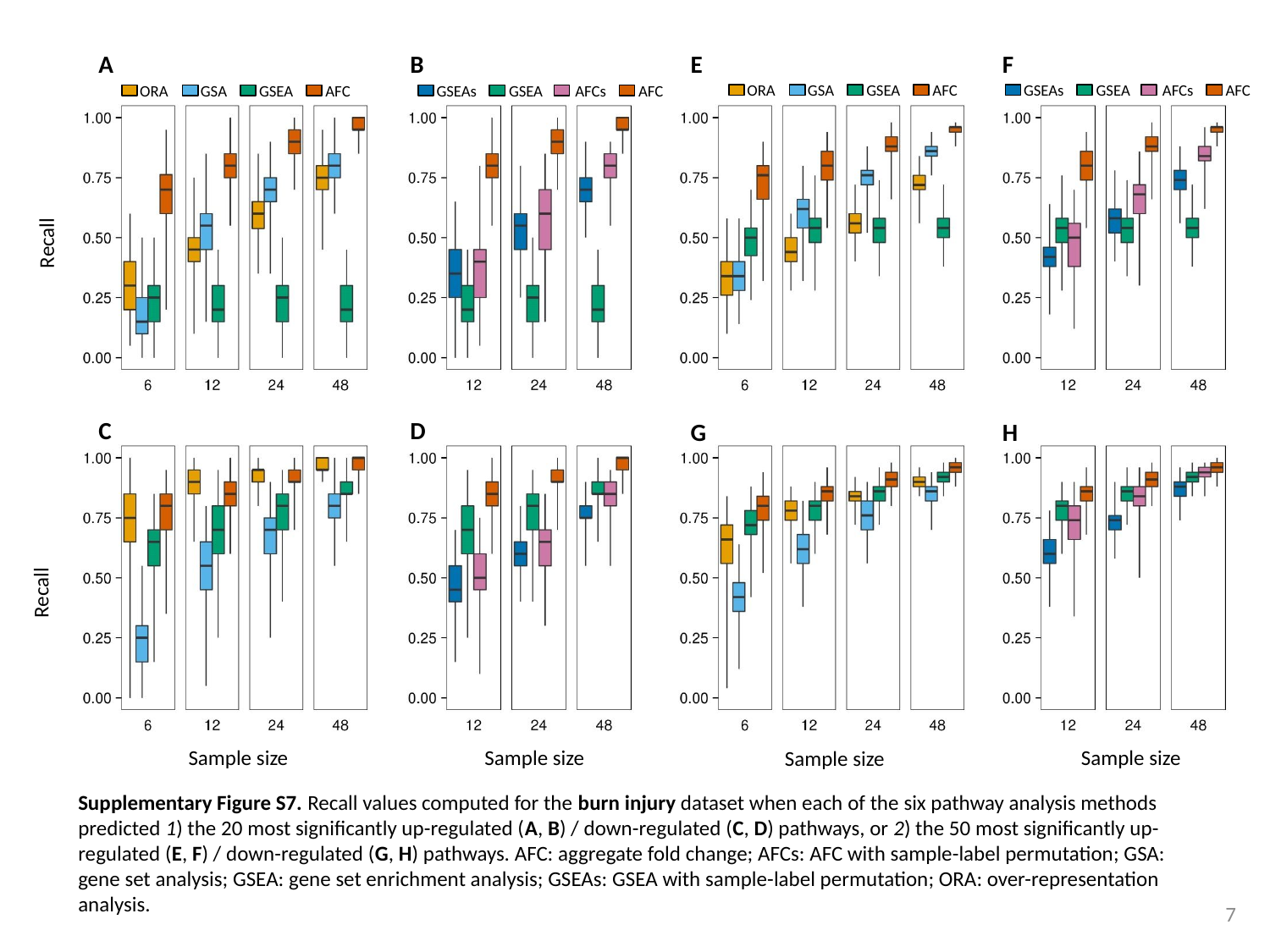

A
B
E
F
ORA GSA GSEA AFC
GSEAs GSEA AFCs AFC
ORA GSA GSEA AFC
GSEAs GSEA AFCs AFC
Recall
C
D
G
H
Recall
Sample size
Sample size
Sample size
Sample size
Supplementary Figure S7. Recall values computed for the burn injury dataset when each of the six pathway analysis methods predicted 1) the 20 most significantly up-regulated (A, B) / down-regulated (C, D) pathways, or 2) the 50 most significantly up-regulated (E, F) / down-regulated (G, H) pathways. AFC: aggregate fold change; AFCs: AFC with sample-label permutation; GSA: gene set analysis; GSEA: gene set enrichment analysis; GSEAs: GSEA with sample-label permutation; ORA: over-representation analysis.
7

## Slide 8
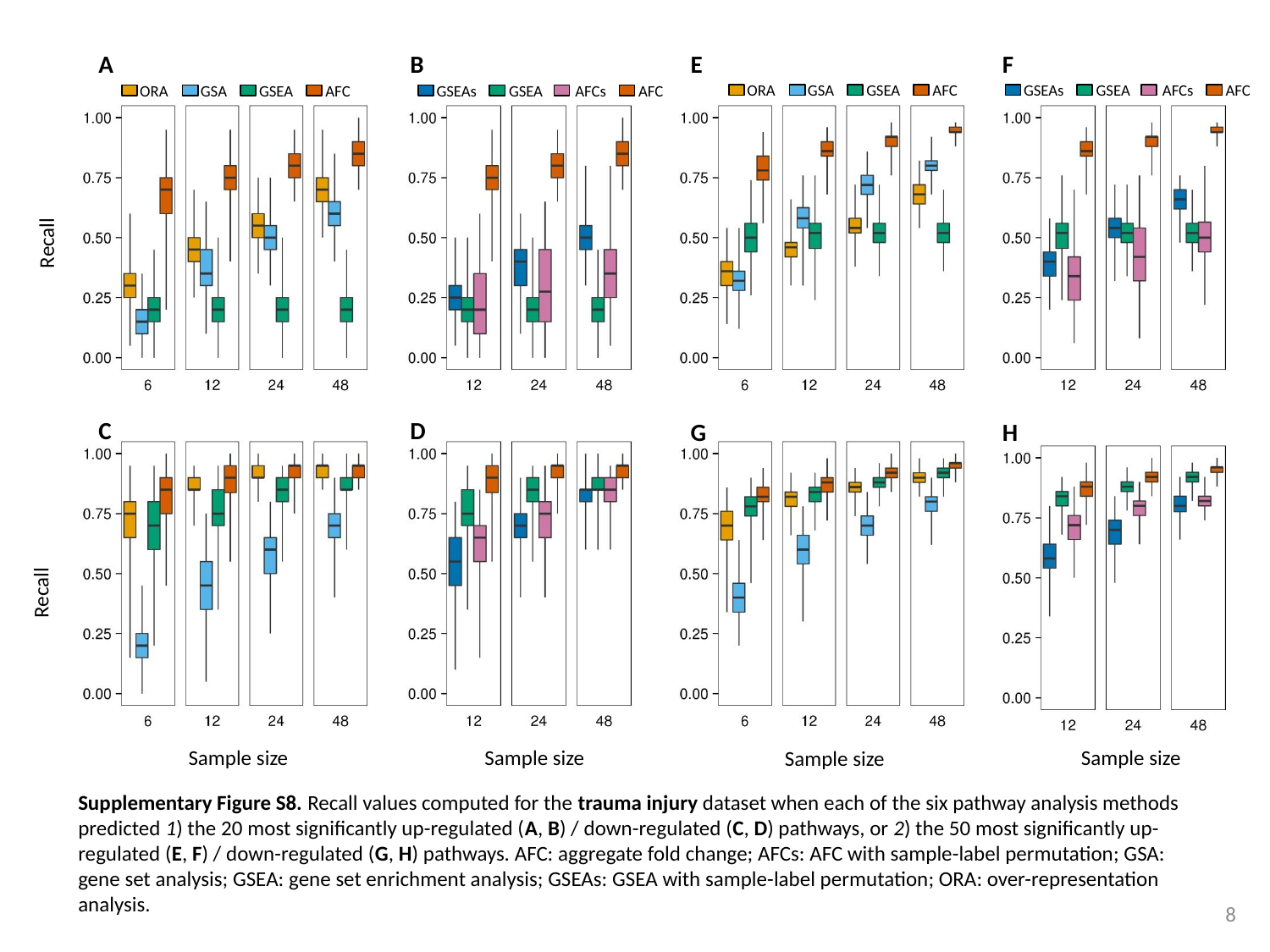

A
B
E
F
ORA GSA GSEA AFC
GSEAs GSEA AFCs AFC
ORA GSA GSEA AFC
GSEAs GSEA AFCs AFC
Recall
C
D
G
H
Recall
Sample size
Sample size
Sample size
Sample size
Supplementary Figure S8. Recall values computed for the trauma injury dataset when each of the six pathway analysis methods predicted 1) the 20 most significantly up-regulated (A, B) / down-regulated (C, D) pathways, or 2) the 50 most significantly up-regulated (E, F) / down-regulated (G, H) pathways. AFC: aggregate fold change; AFCs: AFC with sample-label permutation; GSA: gene set analysis; GSEA: gene set enrichment analysis; GSEAs: GSEA with sample-label permutation; ORA: over-representation analysis.
8

## Slide 9
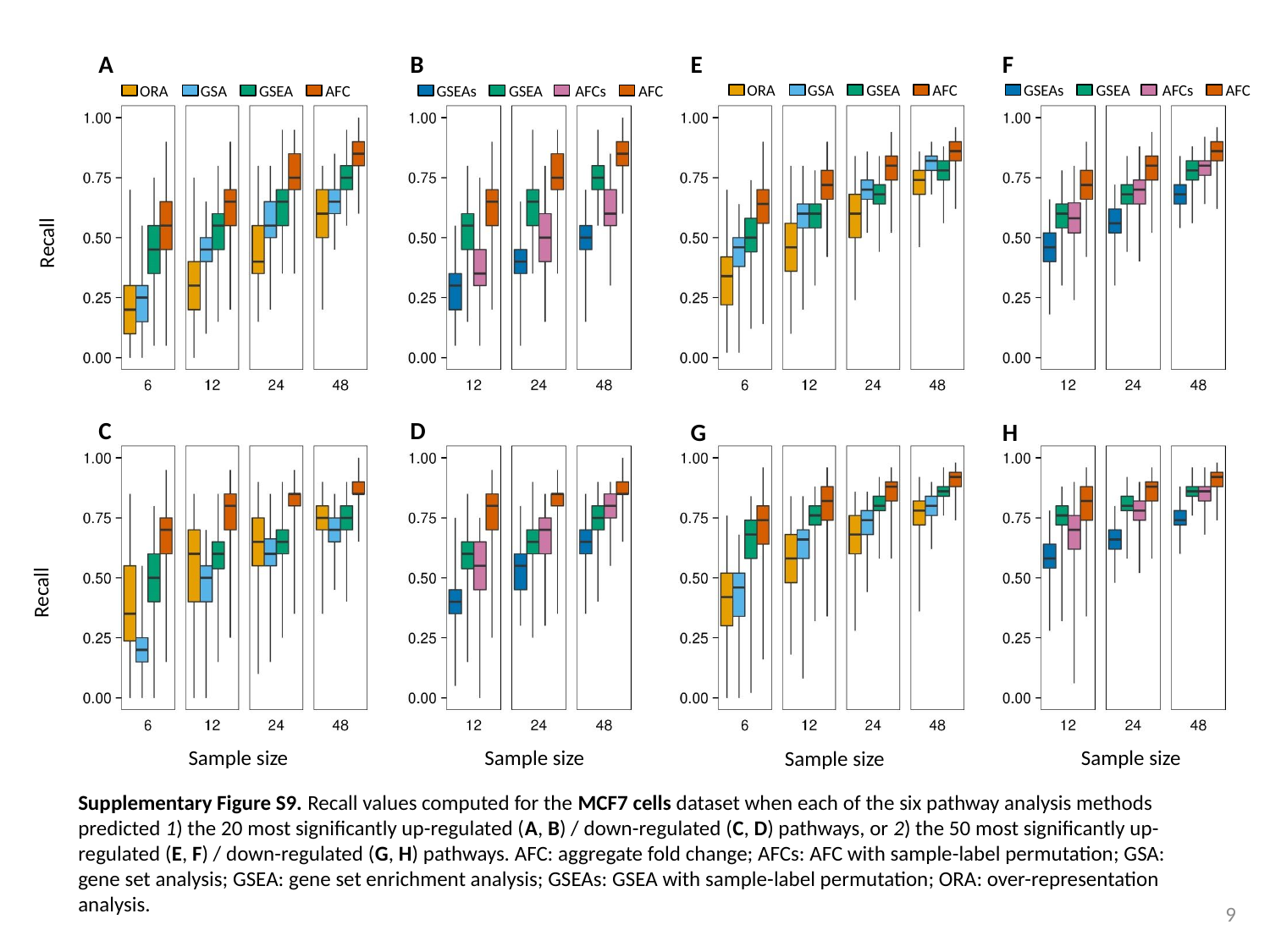

A
B
E
F
ORA GSA GSEA AFC
GSEAs GSEA AFCs AFC
ORA GSA GSEA AFC
GSEAs GSEA AFCs AFC
Recall
C
D
G
H
Recall
Sample size
Sample size
Sample size
Sample size
Supplementary Figure S9. Recall values computed for the MCF7 cells dataset when each of the six pathway analysis methods predicted 1) the 20 most significantly up-regulated (A, B) / down-regulated (C, D) pathways, or 2) the 50 most significantly up-regulated (E, F) / down-regulated (G, H) pathways. AFC: aggregate fold change; AFCs: AFC with sample-label permutation; GSA: gene set analysis; GSEA: gene set enrichment analysis; GSEAs: GSEA with sample-label permutation; ORA: over-representation analysis.
9

## Slide 10
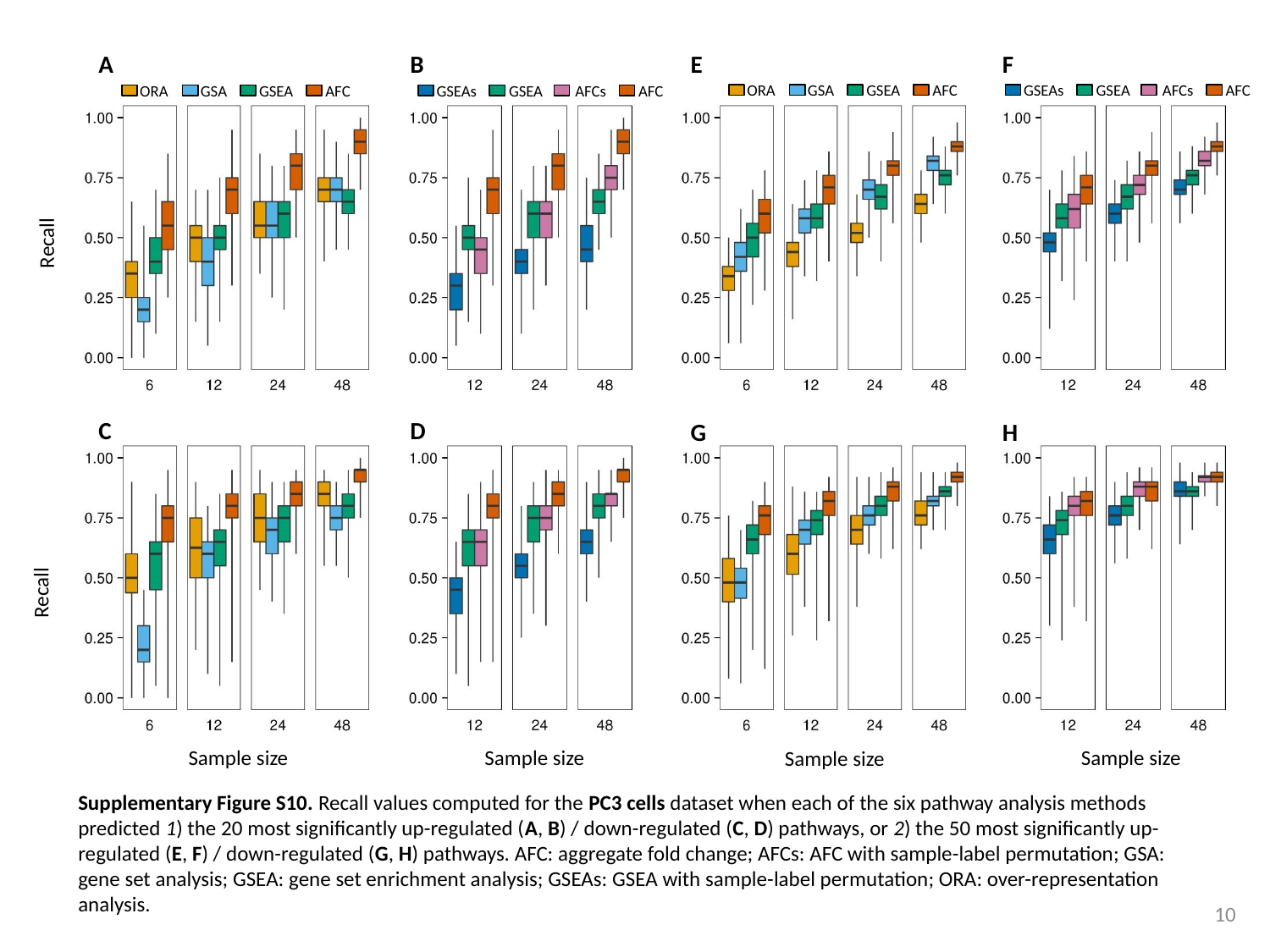

A
B
E
F
ORA GSA GSEA AFC
GSEAs GSEA AFCs AFC
ORA GSA GSEA AFC
GSEAs GSEA AFCs AFC
Recall
C
D
G
H
Recall
Sample size
Sample size
Sample size
Sample size
Supplementary Figure S10. Recall values computed for the PC3 cells dataset when each of the six pathway analysis methods predicted 1) the 20 most significantly up-regulated (A, B) / down-regulated (C, D) pathways, or 2) the 50 most significantly up-regulated (E, F) / down-regulated (G, H) pathways. AFC: aggregate fold change; AFCs: AFC with sample-label permutation; GSA: gene set analysis; GSEA: gene set enrichment analysis; GSEAs: GSEA with sample-label permutation; ORA: over-representation analysis.
10

## Slide 11
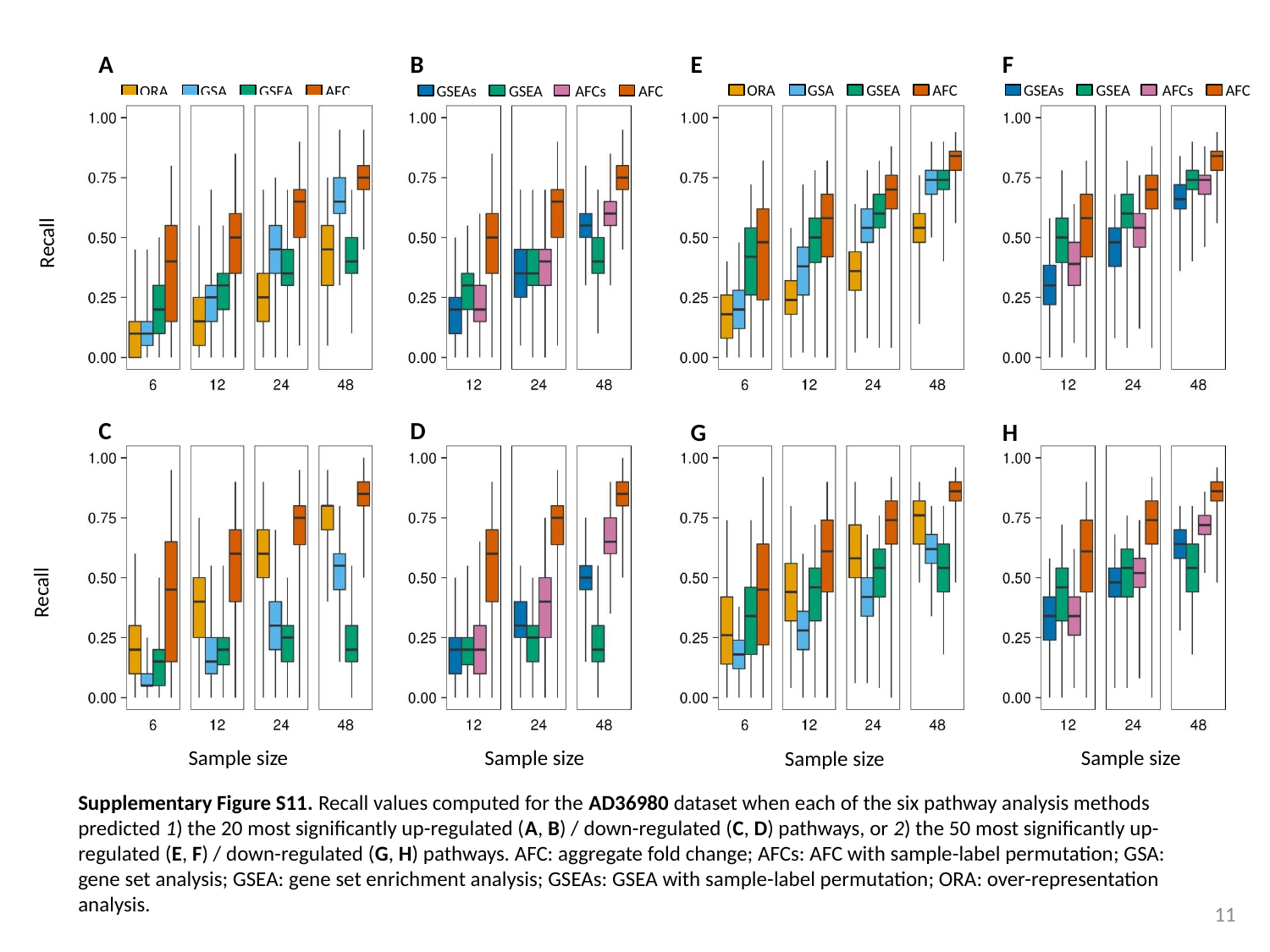

A
B
E
F
ORA GSA GSEA AFC
GSEAs GSEA AFCs AFC
ORA GSA GSEA AFC
GSEAs GSEA AFCs AFC
Recall
C
D
G
H
Recall
Sample size
Sample size
Sample size
Sample size
Supplementary Figure S11. Recall values computed for the AD36980 dataset when each of the six pathway analysis methods predicted 1) the 20 most significantly up-regulated (A, B) / down-regulated (C, D) pathways, or 2) the 50 most significantly up-regulated (E, F) / down-regulated (G, H) pathways. AFC: aggregate fold change; AFCs: AFC with sample-label permutation; GSA: gene set analysis; GSEA: gene set enrichment analysis; GSEAs: GSEA with sample-label permutation; ORA: over-representation analysis.
11

## Slide 12
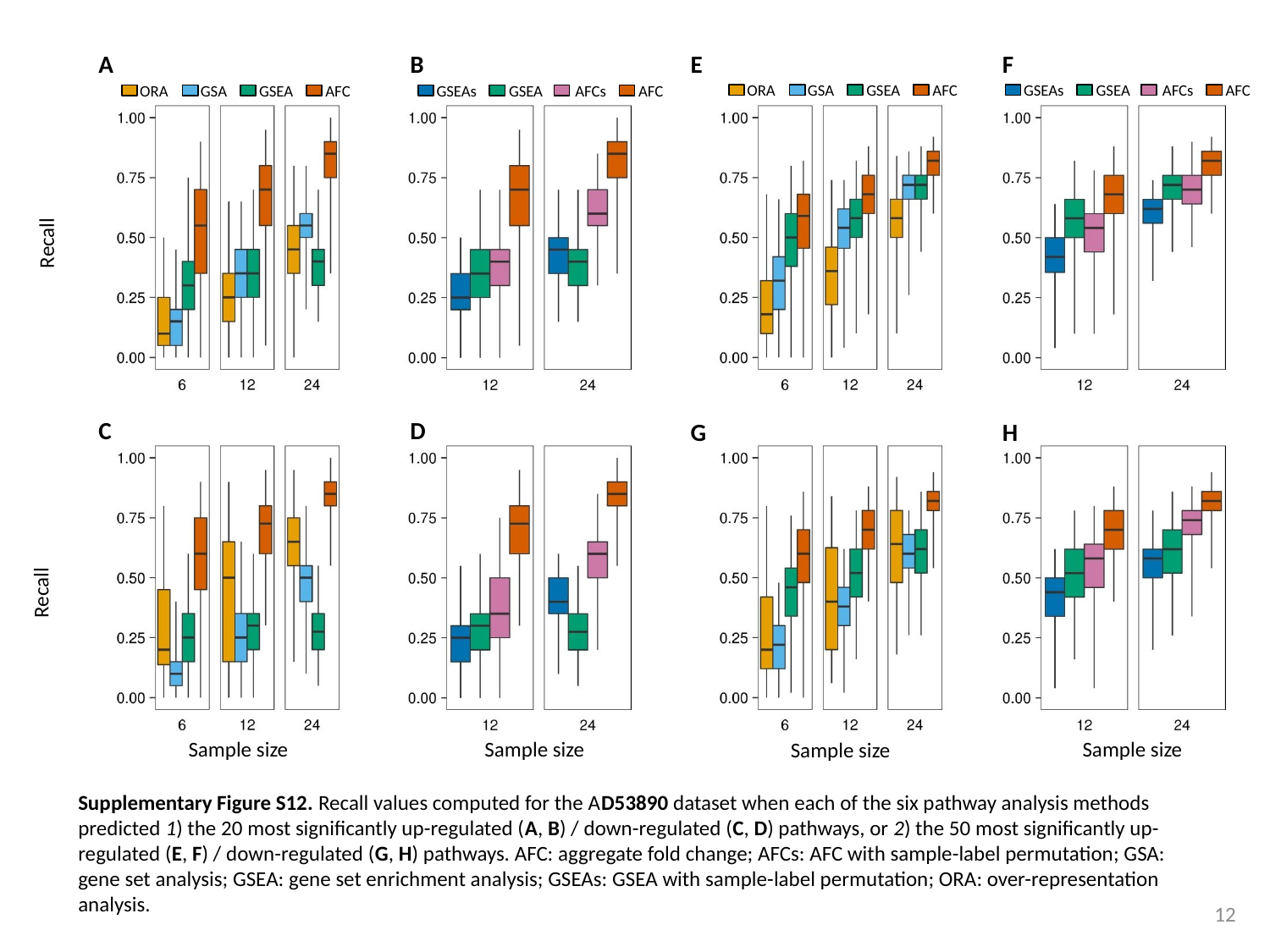

A
B
E
F
ORA GSA GSEA AFC
GSEAs GSEA AFCs AFC
ORA GSA GSEA AFC
GSEAs GSEA AFCs AFC
Recall
C
D
G
H
Recall
Sample size
Sample size
Sample size
Sample size
Supplementary Figure S12. Recall values computed for the AD53890 dataset when each of the six pathway analysis methods predicted 1) the 20 most significantly up-regulated (A, B) / down-regulated (C, D) pathways, or 2) the 50 most significantly up-regulated (E, F) / down-regulated (G, H) pathways. AFC: aggregate fold change; AFCs: AFC with sample-label permutation; GSA: gene set analysis; GSEA: gene set enrichment analysis; GSEAs: GSEA with sample-label permutation; ORA: over-representation analysis.
12

## Slide 13
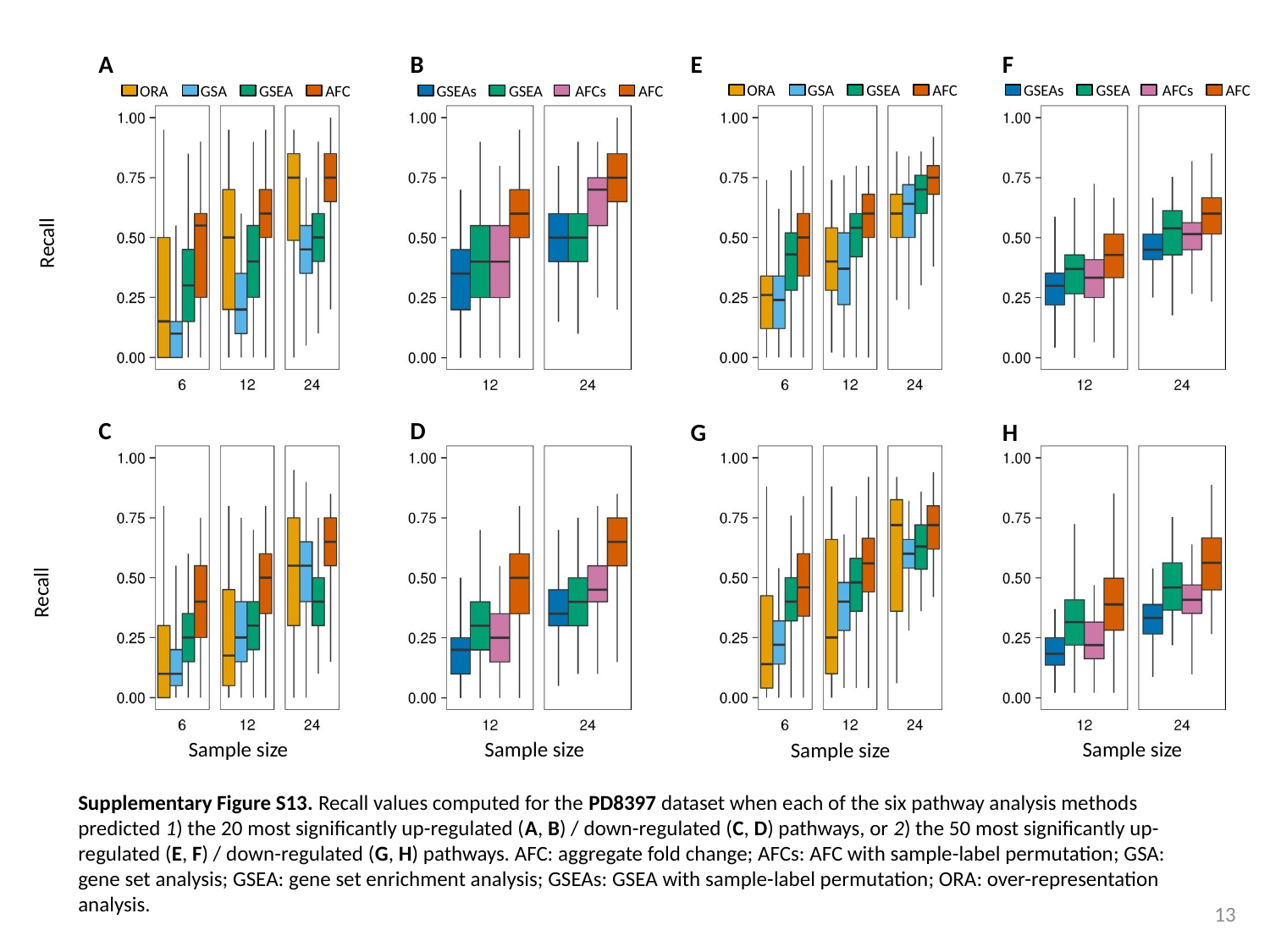

A
B
E
F
ORA GSA GSEA AFC
GSEAs GSEA AFCs AFC
ORA GSA GSEA AFC
GSEAs GSEA AFCs AFC
Recall
C
D
G
H
Recall
Sample size
Sample size
Sample size
Sample size
Supplementary Figure S13. Recall values computed for the PD8397 dataset when each of the six pathway analysis methods predicted 1) the 20 most significantly up-regulated (A, B) / down-regulated (C, D) pathways, or 2) the 50 most significantly up-regulated (E, F) / down-regulated (G, H) pathways. AFC: aggregate fold change; AFCs: AFC with sample-label permutation; GSA: gene set analysis; GSEA: gene set enrichment analysis; GSEAs: GSEA with sample-label permutation; ORA: over-representation analysis.
13

## Slide 14
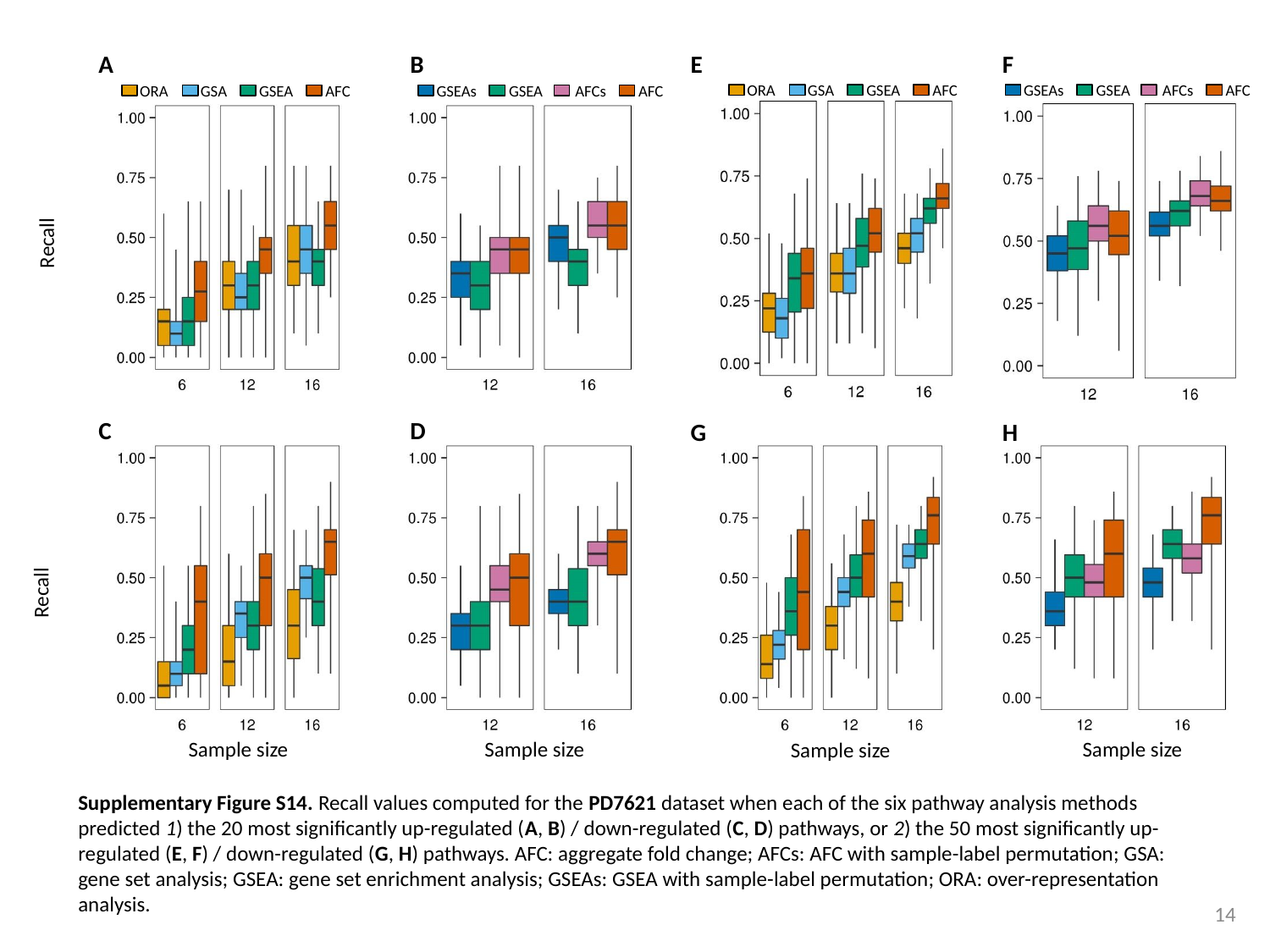

A
B
E
F
ORA GSA GSEA AFC
GSEAs GSEA AFCs AFC
ORA GSA GSEA AFC
GSEAs GSEA AFCs AFC
Recall
C
D
G
H
Recall
Sample size
Sample size
Sample size
Sample size
Supplementary Figure S14. Recall values computed for the PD7621 dataset when each of the six pathway analysis methods predicted 1) the 20 most significantly up-regulated (A, B) / down-regulated (C, D) pathways, or 2) the 50 most significantly up-regulated (E, F) / down-regulated (G, H) pathways. AFC: aggregate fold change; AFCs: AFC with sample-label permutation; GSA: gene set analysis; GSEA: gene set enrichment analysis; GSEAs: GSEA with sample-label permutation; ORA: over-representation analysis.
14

## Slide 15
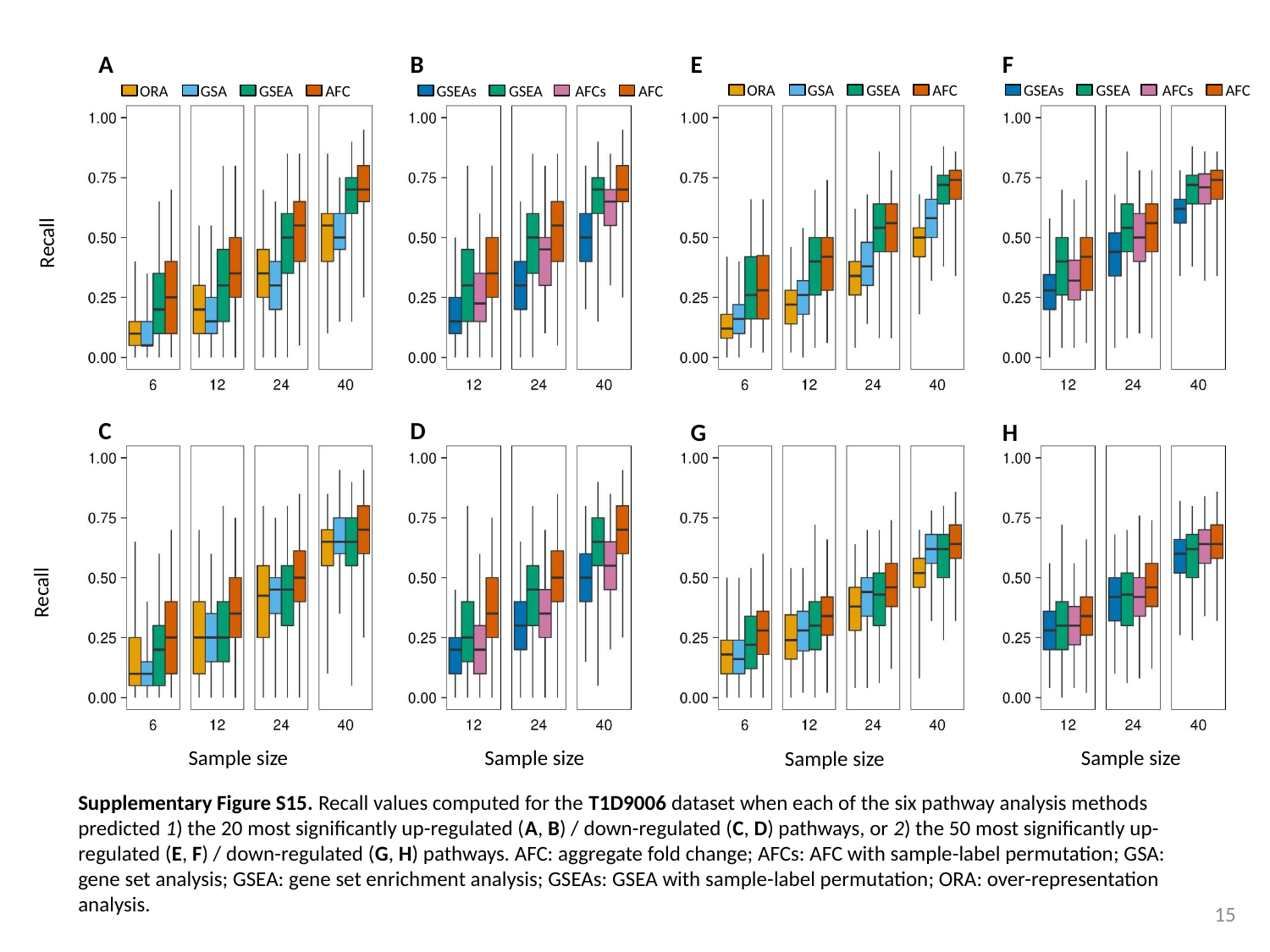

A
B
E
F
ORA GSA GSEA AFC
GSEAs GSEA AFCs AFC
ORA GSA GSEA AFC
GSEAs GSEA AFCs AFC
Recall
C
D
G
H
Recall
Sample size
Sample size
Sample size
Sample size
Supplementary Figure S15. Recall values computed for the T1D9006 dataset when each of the six pathway analysis methods predicted 1) the 20 most significantly up-regulated (A, B) / down-regulated (C, D) pathways, or 2) the 50 most significantly up-regulated (E, F) / down-regulated (G, H) pathways. AFC: aggregate fold change; AFCs: AFC with sample-label permutation; GSA: gene set analysis; GSEA: gene set enrichment analysis; GSEAs: GSEA with sample-label permutation; ORA: over-representation analysis.
15

## Slide 16
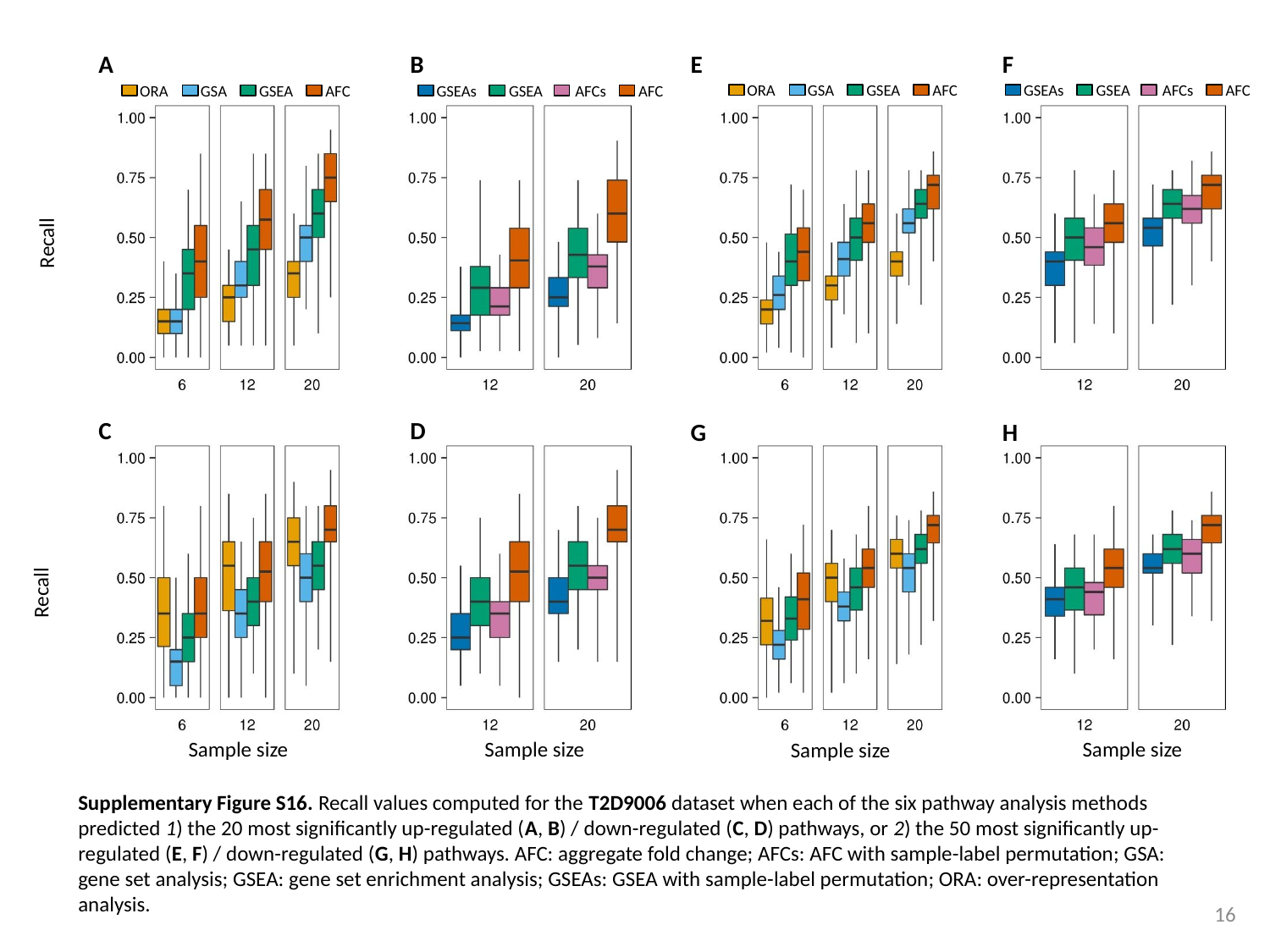

A
B
E
F
ORA GSA GSEA AFC
GSEAs GSEA AFCs AFC
ORA GSA GSEA AFC
GSEAs GSEA AFCs AFC
Recall
C
D
G
H
Recall
Sample size
Sample size
Sample size
Sample size
Supplementary Figure S16. Recall values computed for the T2D9006 dataset when each of the six pathway analysis methods predicted 1) the 20 most significantly up-regulated (A, B) / down-regulated (C, D) pathways, or 2) the 50 most significantly up-regulated (E, F) / down-regulated (G, H) pathways. AFC: aggregate fold change; AFCs: AFC with sample-label permutation; GSA: gene set analysis; GSEA: gene set enrichment analysis; GSEAs: GSEA with sample-label permutation; ORA: over-representation analysis.
16

## Slide 17
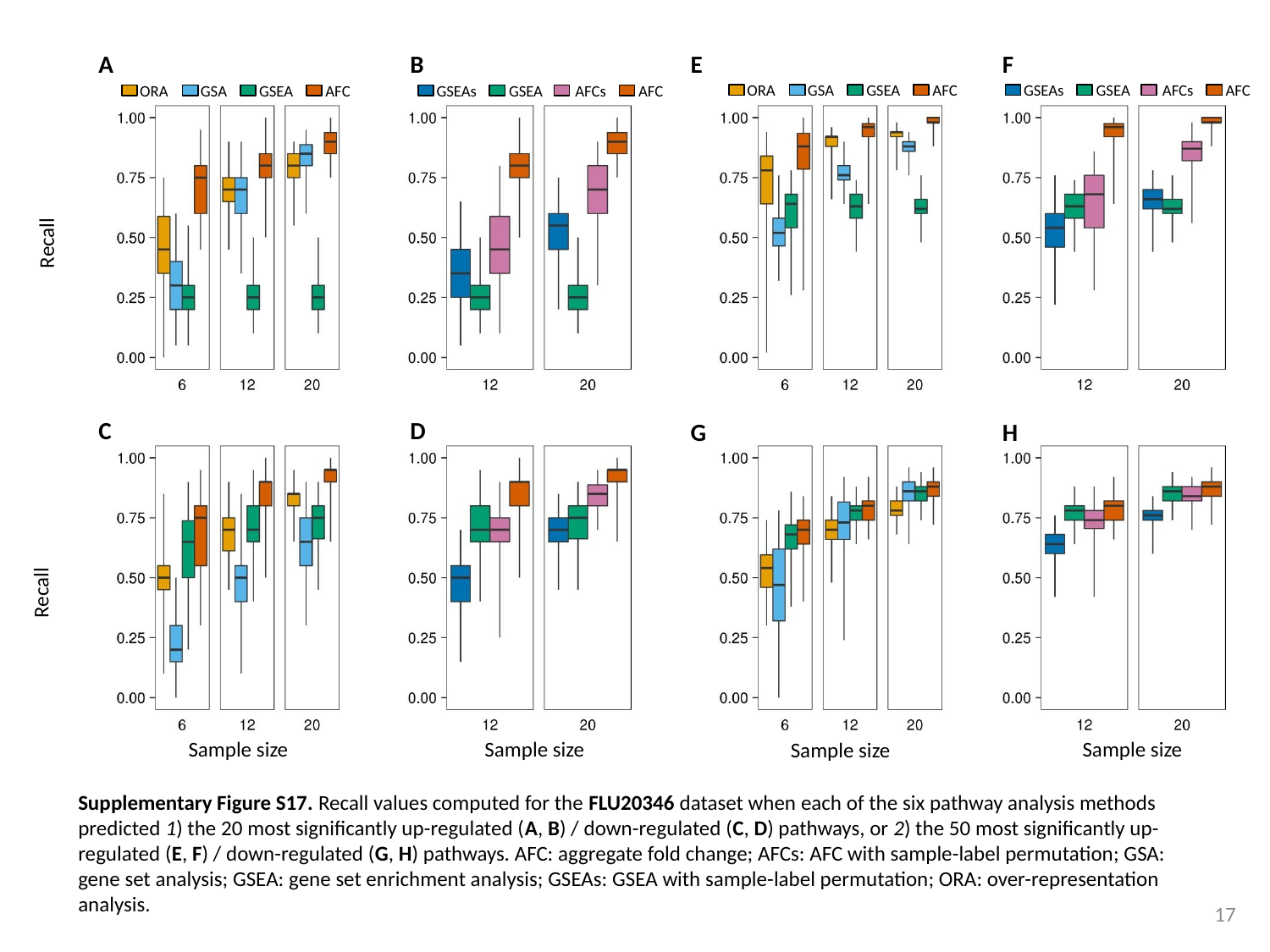

A
B
E
F
ORA GSA GSEA AFC
GSEAs GSEA AFCs AFC
ORA GSA GSEA AFC
GSEAs GSEA AFCs AFC
Recall
C
D
G
H
Recall
Sample size
Sample size
Sample size
Sample size
Supplementary Figure S17. Recall values computed for the FLU20346 dataset when each of the six pathway analysis methods predicted 1) the 20 most significantly up-regulated (A, B) / down-regulated (C, D) pathways, or 2) the 50 most significantly up-regulated (E, F) / down-regulated (G, H) pathways. AFC: aggregate fold change; AFCs: AFC with sample-label permutation; GSA: gene set analysis; GSEA: gene set enrichment analysis; GSEAs: GSEA with sample-label permutation; ORA: over-representation analysis.
17

## Slide 18
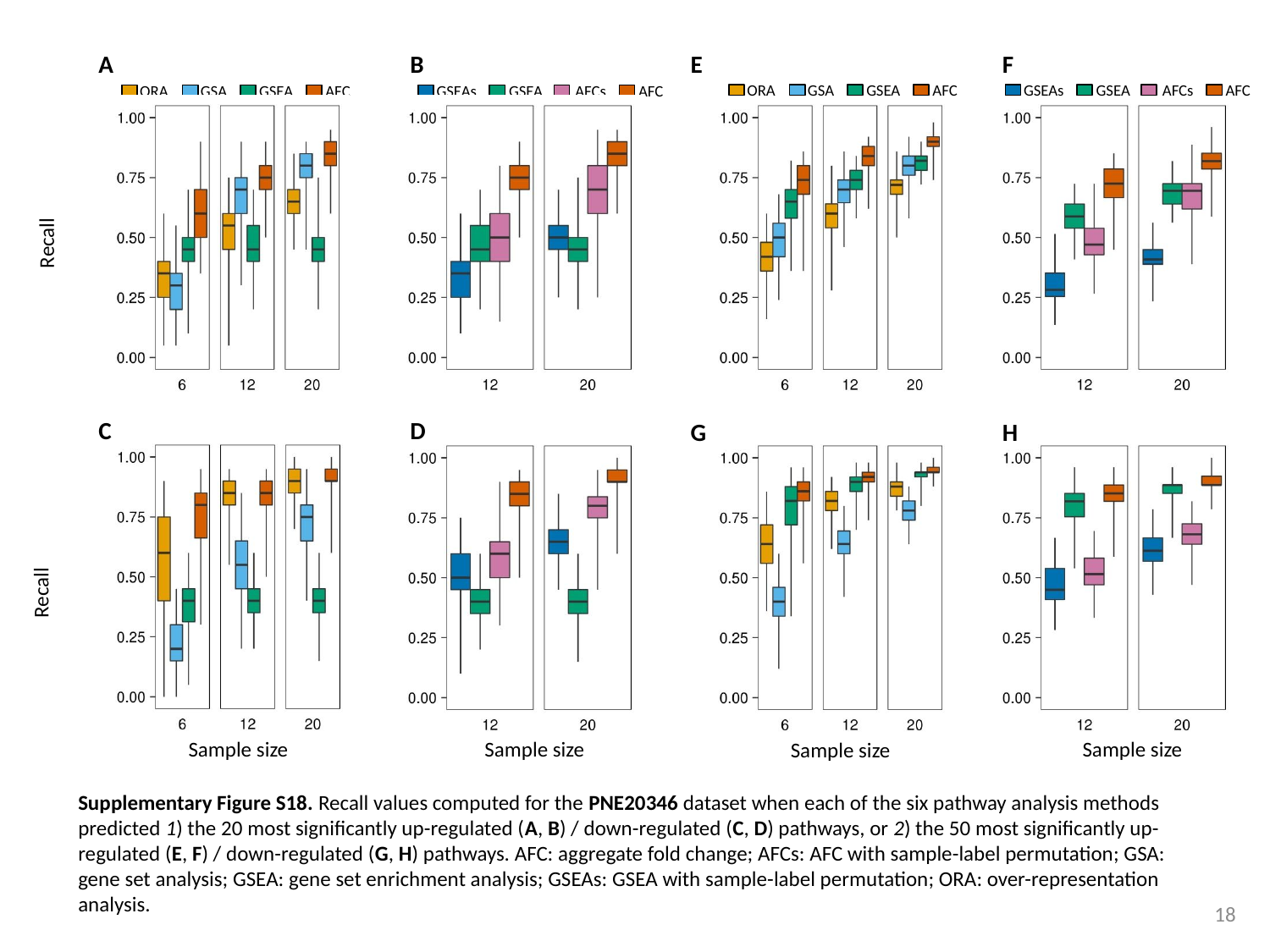

A
B
E
F
ORA GSA GSEA AFC
GSEAs GSEA AFCs AFC
ORA GSA GSEA AFC
GSEAs GSEA AFCs AFC
Recall
C
D
G
H
Recall
Sample size
Sample size
Sample size
Sample size
Supplementary Figure S18. Recall values computed for the PNE20346 dataset when each of the six pathway analysis methods predicted 1) the 20 most significantly up-regulated (A, B) / down-regulated (C, D) pathways, or 2) the 50 most significantly up-regulated (E, F) / down-regulated (G, H) pathways. AFC: aggregate fold change; AFCs: AFC with sample-label permutation; GSA: gene set analysis; GSEA: gene set enrichment analysis; GSEAs: GSEA with sample-label permutation; ORA: over-representation analysis.
18

## Slide 19
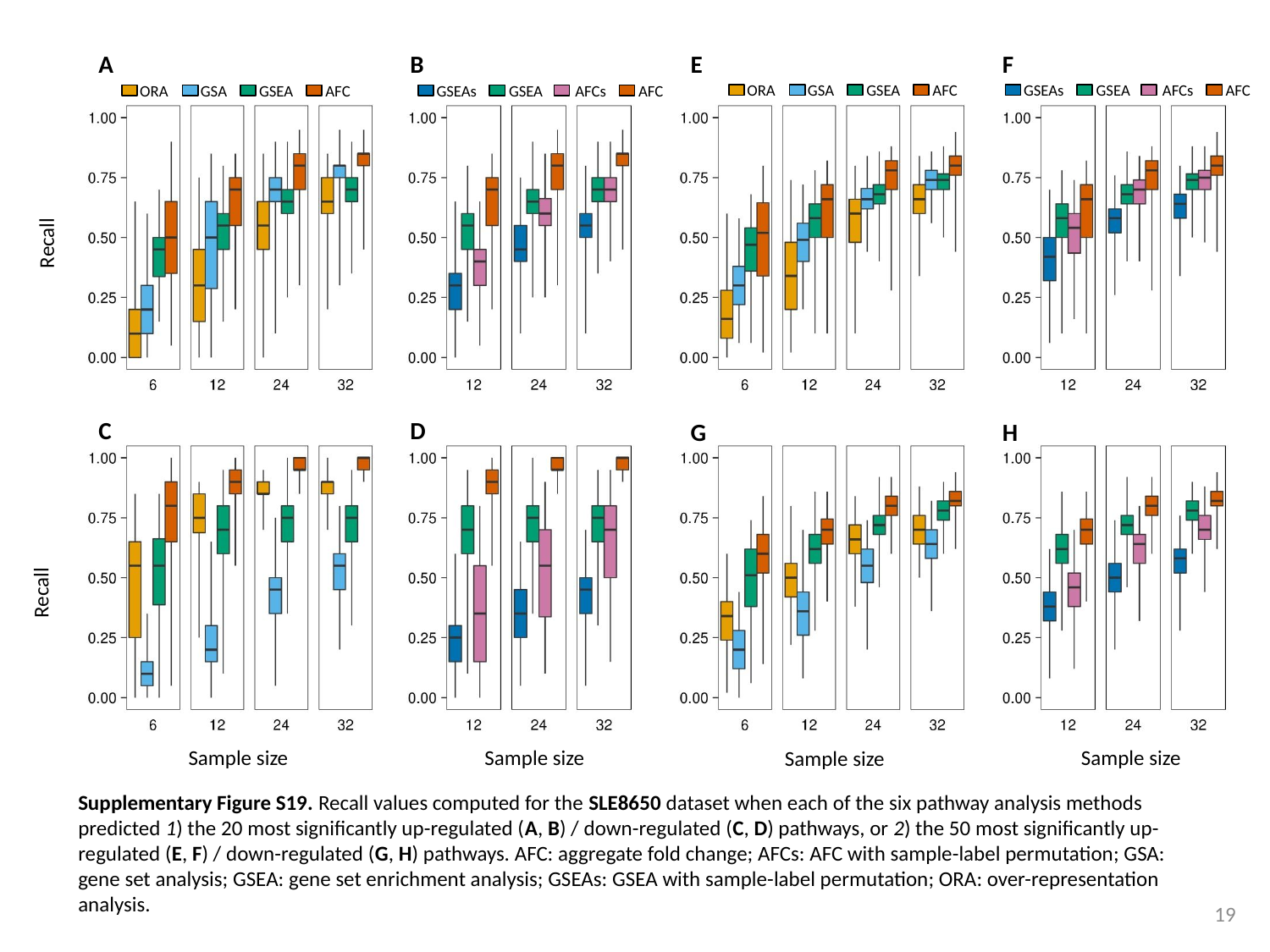

A
B
E
F
ORA GSA GSEA AFC
GSEAs GSEA AFCs AFC
ORA GSA GSEA AFC
GSEAs GSEA AFCs AFC
Recall
C
D
G
H
Recall
Sample size
Sample size
Sample size
Sample size
Supplementary Figure S19. Recall values computed for the SLE8650 dataset when each of the six pathway analysis methods predicted 1) the 20 most significantly up-regulated (A, B) / down-regulated (C, D) pathways, or 2) the 50 most significantly up-regulated (E, F) / down-regulated (G, H) pathways. AFC: aggregate fold change; AFCs: AFC with sample-label permutation; GSA: gene set analysis; GSEA: gene set enrichment analysis; GSEAs: GSEA with sample-label permutation; ORA: over-representation analysis.
19

## Slide 20
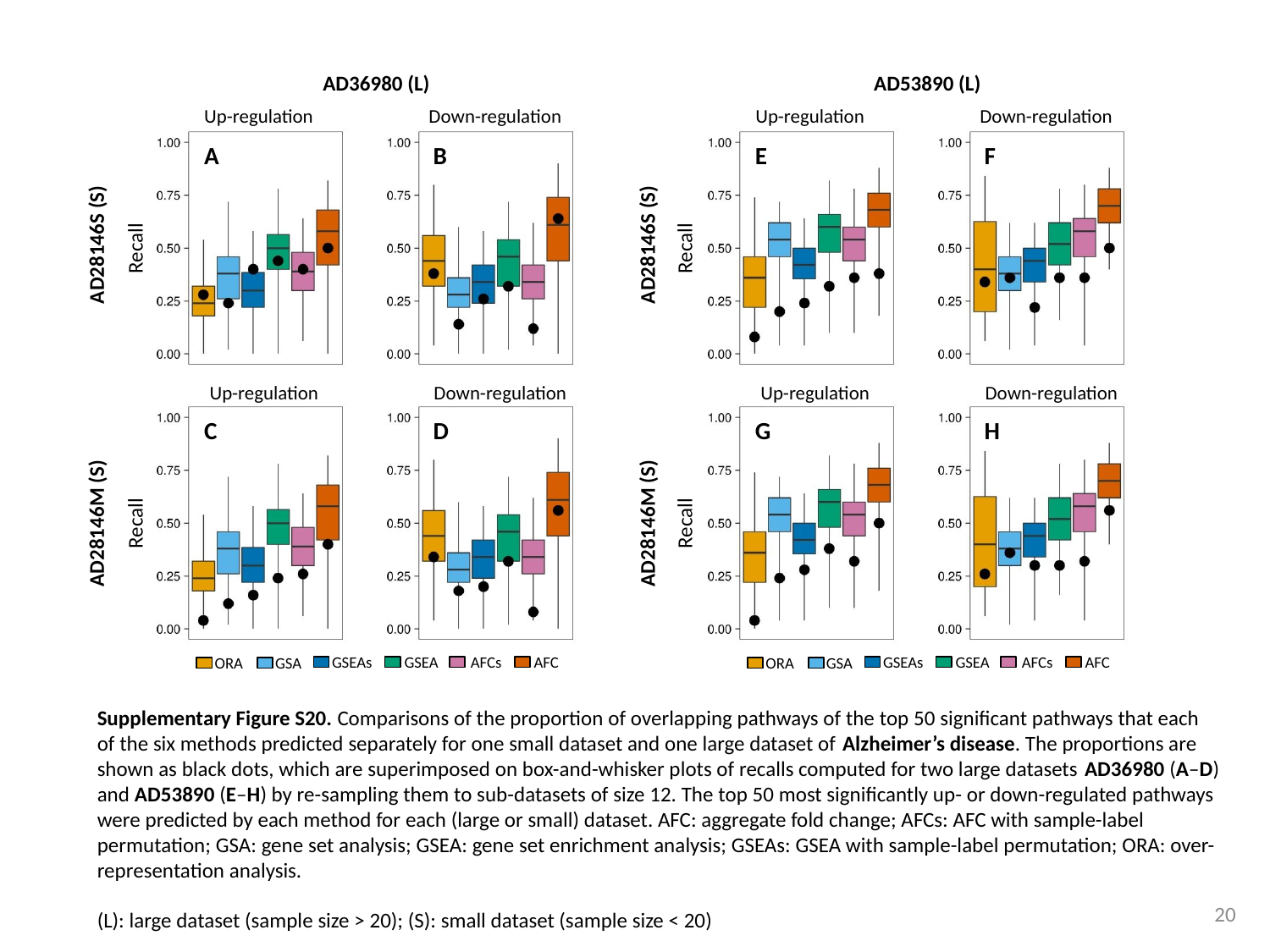

AD36980 (L)
AD53890 (L)
Up-regulation
Down-regulation
Up-regulation
Down-regulation
A
B
E
F
AD28146S (S)
AD28146S (S)
Recall
Recall
Up-regulation
Down-regulation
Up-regulation
Down-regulation
C
D
G
H
Recall
Recall
AD28146M (S)
AD28146M (S)
GSEAs GSEA AFCs AFC
ORA GSA
GSEAs GSEA AFCs AFC
ORA GSA
Supplementary Figure S20. Comparisons of the proportion of overlapping pathways of the top 50 significant pathways that each of the six methods predicted separately for one small dataset and one large dataset of Alzheimer’s disease. The proportions are shown as black dots, which are superimposed on box-and-whisker plots of recalls computed for two large datasets AD36980 (A–D) and AD53890 (E–H) by re-sampling them to sub-datasets of size 12. The top 50 most significantly up- or down-regulated pathways were predicted by each method for each (large or small) dataset. AFC: aggregate fold change; AFCs: AFC with sample-label permutation; GSA: gene set analysis; GSEA: gene set enrichment analysis; GSEAs: GSEA with sample-label permutation; ORA: over-representation analysis.
(L): large dataset (sample size > 20); (S): small dataset (sample size < 20)
20

## Slide 21
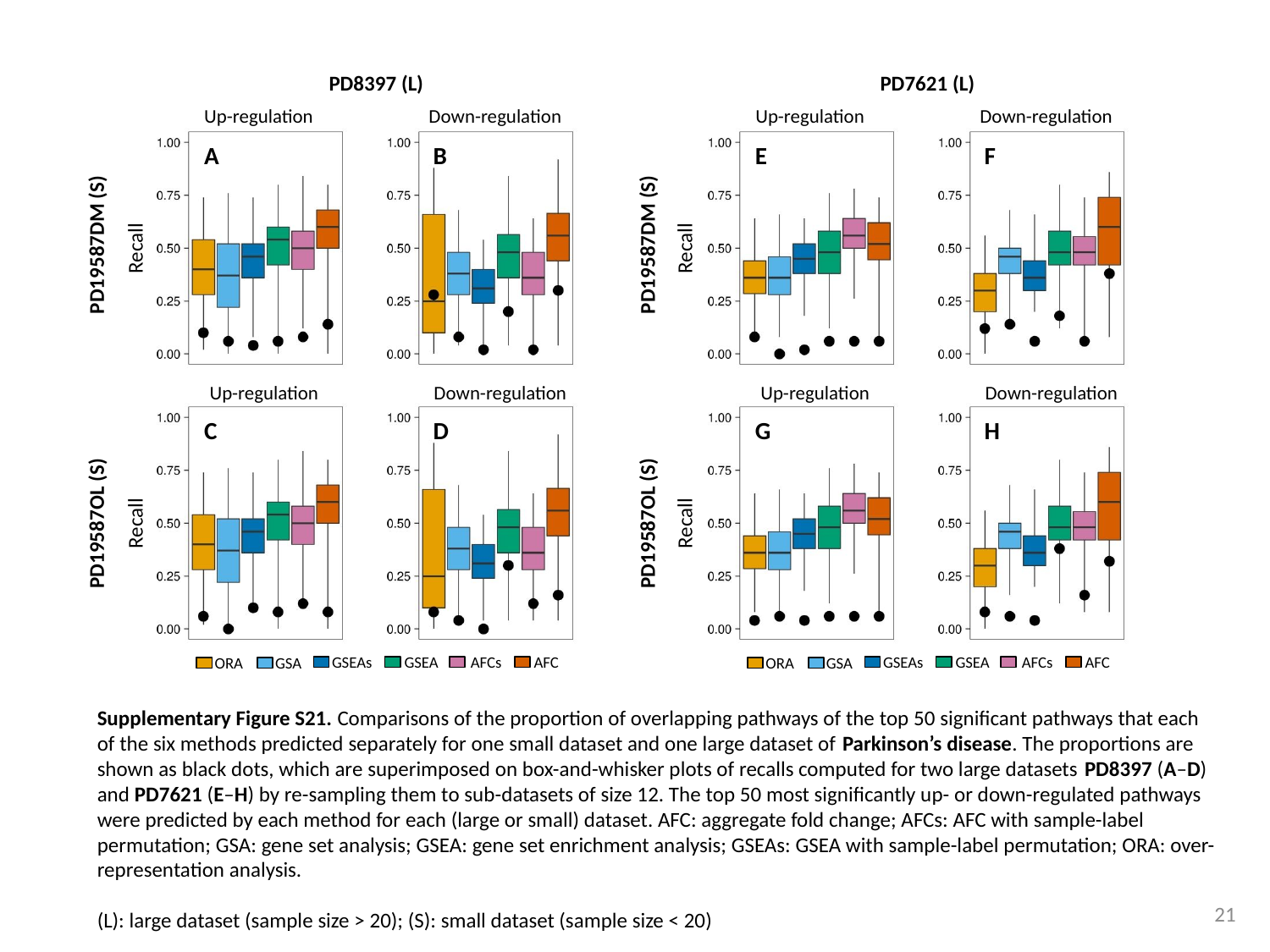

PD8397 (L)
PD7621 (L)
Up-regulation
Down-regulation
Up-regulation
Down-regulation
A
B
E
F
PD19587DM (S)
PD19587DM (S)
Recall
Recall
Up-regulation
Down-regulation
Up-regulation
Down-regulation
C
D
G
H
Recall
Recall
PD19587OL (S)
PD19587OL (S)
GSEAs GSEA AFCs AFC
ORA GSA
GSEAs GSEA AFCs AFC
ORA GSA
Supplementary Figure S21. Comparisons of the proportion of overlapping pathways of the top 50 significant pathways that each of the six methods predicted separately for one small dataset and one large dataset of Parkinson’s disease. The proportions are shown as black dots, which are superimposed on box-and-whisker plots of recalls computed for two large datasets PD8397 (A–D) and PD7621 (E–H) by re-sampling them to sub-datasets of size 12. The top 50 most significantly up- or down-regulated pathways were predicted by each method for each (large or small) dataset. AFC: aggregate fold change; AFCs: AFC with sample-label permutation; GSA: gene set analysis; GSEA: gene set enrichment analysis; GSEAs: GSEA with sample-label permutation; ORA: over-representation analysis.
(L): large dataset (sample size > 20); (S): small dataset (sample size < 20)
21

## Slide 22
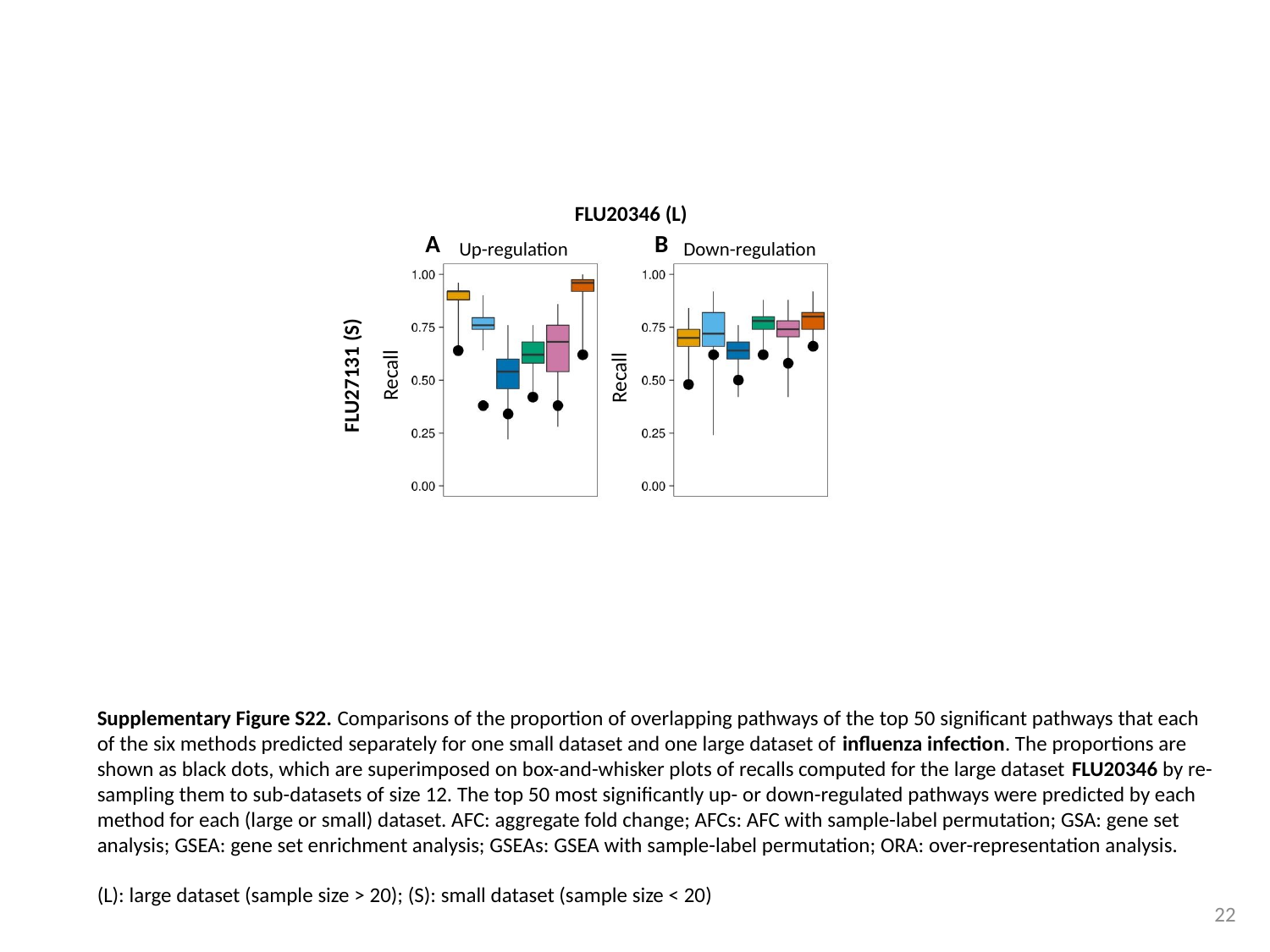

FLU20346 (L)
A
B
Up-regulation
Down-regulation
FLU27131 (S)
Recall
Recall
Supplementary Figure S22. Comparisons of the proportion of overlapping pathways of the top 50 significant pathways that each of the six methods predicted separately for one small dataset and one large dataset of influenza infection. The proportions are shown as black dots, which are superimposed on box-and-whisker plots of recalls computed for the large dataset FLU20346 by re-sampling them to sub-datasets of size 12. The top 50 most significantly up- or down-regulated pathways were predicted by each method for each (large or small) dataset. AFC: aggregate fold change; AFCs: AFC with sample-label permutation; GSA: gene set analysis; GSEA: gene set enrichment analysis; GSEAs: GSEA with sample-label permutation; ORA: over-representation analysis.
(L): large dataset (sample size > 20); (S): small dataset (sample size < 20)
22
